# Genetic Organization of Interphase Chromosome Bands and Interbands in *Drosophila melanogaster*

**DOI:** 10.1371/journal.pone.0101631

**Published:** 2014-07-29

**Authors:** Igor F. Zhimulev, Tatyana Yu. Zykova, Fyodor P. Goncharov, Varvara A. Khoroshko, Olga V. Demakova, Valeriy F. Semeshin, Galina V. Pokholkova, Lidiya V. Boldyreva, Darya S. Demidova, Vladimir N. Babenko, Sergey A. Demakov, Elena S. Belyaeva

**Affiliations:** 1 Institute of Molecular and Cellular Biology of the Siberian Branch of the Russian Academy of Sciences, Novosibirsk, Russia; 2 Novosibirsk State University, Novosibirsk, Russia; 3 Institute of Cytology and Genetics of the Siberian Branch of the Russian Academy of Sciences, Novosibirsk, Russia; Virginia Tech, United States of America

## Abstract

*Drosophila melanogaster* polytene chromosomes display specific banding pattern; the underlying genetic organization of this pattern has remained elusive for many years. In the present paper, we analyze 32 cytology-mapped polytene chromosome interbands. We estimated molecular locations of these interbands, described their molecular and genetic organization and demonstrate that polytene chromosome interbands contain the 5′ ends of housekeeping genes. As a rule, interbands display preferential “head-to-head” orientation of genes. They are enriched for “broad” class promoters characteristic of housekeeping genes and associate with open chromatin proteins and Origin Recognition Complex (ORC) components. In two regions, 10A and 100B, coding sequences of genes whose 5′-ends reside in interbands map to constantly loosely compacted, early-replicating, so-called “grey” bands. Comparison of expression patterns of genes mapping to late-replicating dense bands vs genes whose promoter regions map to interbands shows that the former are generally tissue-specific, whereas the latter are represented by ubiquitously active genes. Analysis of RNA-seq data (modENCODE-FlyBase) indicates that transcripts from interband-mapping genes are present in most tissues and cell lines studied, across most developmental stages and upon various treatment conditions. We developed a special algorithm to computationally process protein localization data generated by the modENCODE project and show that *Drosophila* genome has about 5700 sites that demonstrate all the features shared by the interbands cytologically mapped to date.

## Introduction

*Drosophila* polytene chromosomes have served as the best available model of eukaryotic interphase chromosome. They are prominent for their banding pattern formed by dark transverse stripes (called bands), which encompass large chunks of chromatin material. These bands alternate with fine, lighter-colored stripes that have less material and are more loosely packed. Such light-transparent structures between bands are known as interbands.

Genetic organization of bands and interbands defined as the pattern that sets positioning of genes and genetic features relatively to the structural elements of a chromosome, is still largely elusive. This is due to the fact that despite the availability of the *Drosophila* genome, methods to even approximately map band/interband borders on a physical map are still lacking.

Yet, many interesting hypotheses regarding the genetic organization of bands and interbands in polytene chromosomes have been put forth. Some of the points of these hypotheses were experimentally validated, so we believe it is important to consider them below.

Genes were proposed to reside in interbands [1], or bands (1–2 genes/band) [2]–[5]. Also, bands were proposed to contain many structural genes transcribed coordinately and as polycistronic messages [6]. In some models, band and interband were considered to form a single genetic unit, where one part of the gene was embedded in a band, and the other part mapped to an interband [7]–[12]. The Paul model [8] is of special interest. The author considered interband regions as essentially polymerase-binding sites, so transcription would progress into band regions from initiation sites which were likely situated near the band-interband junctions.

Very interesting conclusions were made regarding the general meaning of banding pattern: bands were regarded as hosting inactivated genes, interbands were represented by the genes in a steady state of activity [13]–[15]; in other models, interbands were believed to contain constantly active housekeeping genes [16]–[17]. Further details on various banding pattern models are available in [18].

Several recent technological advances have dramatically moved forward our understanding of polytene chromosome organization. First, efforts of the modENCODE project have produced genome-wide profiling data for many proteins that specifically localized to bands or interbands in interphase chromosomes ([19], for review).

Secondly, using genome-wide DamID mapping of 53 chromosomal proteins and histone modifications Filion et al. [20] have generated a map of *Drosophila* chromatin landscape and demonstrated that the genome can be segmented into five main chromatin types. Conditionally named “BLUE” (Pc-dependent repression) and “BLACK” (repression mechanism not defined) chromatin types associated with repressed chromatin, “YELLOW” chromatin contained ubiquitously expressed genes, whereas “RED” chromatin harbored active genes with more complex expression patterns. “GREEN” chromatin type was defined by enrichment of heterochromatin-specific proteins HP1 and Su(var)3–9 ([21], for discussion). More refined analysis of modENCODE data resulted in description of many more chromatin states [22]. Significant proportion of genome sequence is known to map to a special class of polytene chromosome bands, called intercalary heterochromatin (IH) [23]. These chromosome regions associate with BLACK chromatin proteins (H1, SUUR, LAM, D1) and range from 100 to 700 kb in length. Here, DNA replicates late and compared to the genome average, these regions have lower gene density [24], [25]. Genomic localization of proteins that constitute repressed chromatin can thus be used as a marker to establish the molecular position of IH [26].

Third, we recently developed an approach to simultaneously map the interband material on polytene chromosomes and in the genome using transposon insertion tags. This allows exact localization of insertion sites both on cytological and physical maps as well as precise identification of sequences around the transposon integration sites.

Using this approach, we describe protein composition and other chromatin parameters in 12 DNA sequences corresponding to polytene chromosome interbands. They display general features of open chromatin: low nucleosome density, histone H1 dips, association with TSS-specific proteins such as RNA polymerase II, various transcription factors, nucleosome remodeling proteins - NURF, ISWI, WDS, interband-specific proteins (CHRIZ/CHROMATOR, CHRIZ hereafter), proteins of origin recognition complexes (ORC). Moreover, they show clustering of DNaseI hypersensitive sites (DHS) (see for discussion [27], [28]).

Based on these data, we subdivided all polytene chromosome bands into two contrasting groups: loosely compacted early-replicating, so-called “grey” bands and dense late-replicating compact bands (“black” IH bands). They differ in many aspects of their protein and genetic make-up, as well as in DNA compactization [27].

Previously, we showed that polytene chromosomes and interphase chromosomes from dividing cells display identical organization. Namely, interbands from polytene chromosomes and the corresponding DNA sequences from cell line chromosomes share similar features in terms of localization of open chromatin-type proteins. Consequently, banding pattern appears as a fundamental organization principle of interphase chromosomes. In both types of chromosomes, homologous interbands and bands have identical physical borders and length; importantly, they also associate with identical sets of proteins [19], [26], [27]. Hence, the notion of an interband defined as a decondensed region in the context of polytene chromosomes is also applicable to other types of interphase chromosomes. In other words, the term “interband” should be viewed as an equivalent of a constantly decondensed region in the context of any interphase chromosome. Accordingly, hereafter we use this wider definition of an interband.

In the present work, using various cytological approaches, we first characterized a new set of precisely mapped interbands, and then processed the modENCODE data on localization of active chromatin proteins using a custom-designed computation model. This analysis suggests that interphase chromosome interbands contain constantly active promoter regions of ubiquitously active genes. Coding sequences of these genes, at least in two regions studied, map to adjacent loosely compacted early-replicating “grey” bands. In contrast, densely packed, late-replicating bands of polytene chromosomes appear to preferentially harbor tissue-specific genes.

## Results

### Mapping interbands in polytene chromosomes and on a physical map

To analyze the interbands' protein make-up and to explore their molecular organization, these structures must be accurately mapped on both cytological, electron microscopy (EM) and physical maps. In this study, we present the molecular-genetic analysis of a set of interbands (32 in total), which we believe were unambiguously identified at the cytology level; 21 of these interbands were mapped earlier [29]–[33].

A group of 11 interbands was characterized in detail here, by comparing Bridges' polytene maps, EM data, modENCODE protein localization data and mapping of IH regions [26]. This group comprises the interbands from regions 7F, 19E, 21D, 35D, 56A, 58A, 70A, and 100B (see below and Text S1 [35]–[38]).

For illustrative purposes, below we provide detailed description of mapping data for interband regions found in 7F1-2 and 100B.

**Figure 1:**
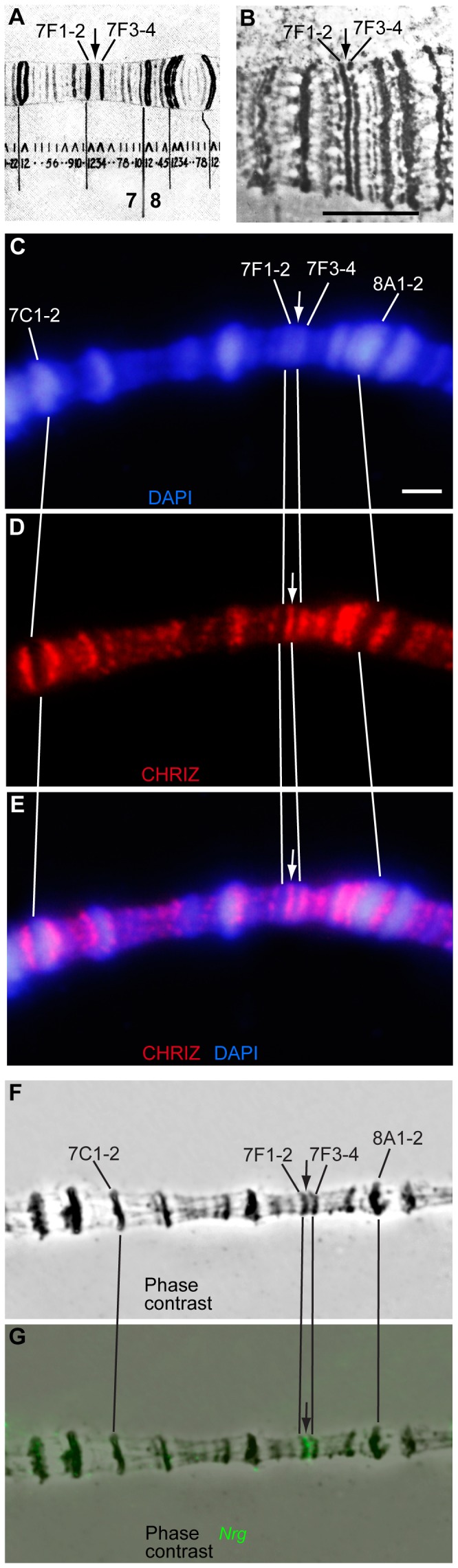
Cytological mapping of an interband (arrow) in the 7F1-4 region of the X chromosome. **A, B** – Fragment of the revised Bridges' map of *D. melanogaster* X chromosome [35] (**A**) and EM image of the region (**B**). Bar corresponds to 2 mkm. **C-E –** immunofluorescence localization of CHRIZ (red) in the interband 7F1-2/7F3-4. DNA is counterstained with DAPI (blue). **F-G –** FISH localization of the DNA probe containing the 5′-fragment of *Nrg* (green in **G**).

In the region 7F, two condensed and late-replicating bands 7F1-2 and 7F3-4 [34] flank a thin and well-defined interband, which is clearly observable both on light microscopy [35] and EM maps. When performing high-resolution analysis of chromosome banding pattern, C. Bridges never reported any additional minibands between these bands (Fig. 1A). Likewise, upon EM analysis of this region, we also observed no additional minibands (the interband of interest is marked by an arrow in Fig. 1 B). This interband is clearly decorated by an interband-specific protein CHRIZ (arrows in Fig. 1C-E), and FISH analysis indicates it harbors the 5′-end of the *Nrg* gene (arrows in Fig. 1F, G).

In the region 100B, polytene maps [39] show two doublets, 100B1-2 and 100B4-5, as well as a very faint band 100B3 in between (Fig. S1A, D). Thus, this region encompasses two interbands, one proximal and one distal to 100B3. DNA material between the bands 100B3 and 100B4-5 hosts the 5′-end of *dco* gene (Fig. S1E, F), whereas the region between 100B1-2 and 100B3 harbors the 5′-end of the gene *l(3)03670* (See Fig. S3, below). Both interbands appear CHRIZ-positive: the region demonstrates two stripes, one located in the interband 100B1-2/100B3 and the other in the 100B3/100B4-5 interband (Fig. S1B, C).

### Molecular and genetic organization of interbands and bands in *Drosophila* chromosomes

#### Algorithm to identify genomic regions enriched in interband chromatin proteins

Earlier we reported high similarity of banding patterns in both polytene and non-polytene diploid cells [19], [27]. Out of the proteins mapped by modENCODE (modENCODE Consortium, 2010) in Kc, S2 and BG3 cell lines, we identified a set of proteins enriched in the regions of 12 reference P-element insertions in the interband regions of polytene chromosomes [27], [28].

In this study, we aimed to partition the whole genome into discrete chromatin states defined by the local enrichment of “open chromatin” proteins that are found predominantly in interbands. To do so, we developed an algorithm that would allow us to assign molecular coordinates to the regions corresponding to interbands of polytene chromosomes, based on the localization of the above-mentioned “interband”-specific protein markers. Additionally, in contrast to the whole-genome approaches [20], [22], our chromatin clustering analysis is based on the combination of protein profiling data across four cell lines: S2, Kc, BG3, Clone 8 using fly modENCODE non-histone protein dataset as a source (modENCODE Consortium, 2010). This was done in order to identify the genomic regions that are generally co-occupied by most of the proteins analyzed.

We first performed correlation analysis of protein localization data generated by the fly modENCODE project, and then proceeded to hierarchical clustering of proteins using the X chromosome as the best characterized chromosome in *Drosophila* genome (dendrogram in Fig. S2). Stopping rule a_i+1_≥a+2s_a_ was used to define the appropriate sensitivity threshold [40], which produced four major classes of proteins. We chose to focus on just one class comprising 12 chromatin proteins, many of which overlapped with the set of interband proteins studied previously (RNA polymerase II, CHRIZ, dMi-2, NURF301, WDS) [27], [28], [41]. Additionally, the cluster included ISWI, JIL-1, MLE, MOF, MRG15, MSL-1 and MBD. Within this cluster, we observed the highest correlation between datasets (see the green frame on the Fig. S2). In order to identify the DNA regions where 12 selected chromatin proteins would preferentially co-localize, we applied principle component analysis (PCA). Closer inspection of the two first principle components (PC1 and PC2) covering 62.4% of the sample variance showed that they scored high in the 12 interband regions described previously [27], particularly, in gene promoter regions (PC1) and in decompacted chromatin regions studied (PC2). To define the tentative interband borders based on PC1 and PC2, we proceeded to the HMM (hidden Markov model) analysis.

Specifically, we performed a series of PC1 and PC2 clustering and allowed the number of states to vary from 2 to 15. When conditioning that all 12 interbands mapped previously consistently group together, a 4-state model was produced. Furthermore, we obtained an independent estimate of clustering quality using Calinski-Harabasz criterion, which similarly returned 4 states (further details are provided in Materials and Methods).

Thus, the genome was partitioned into 4 states. Of these, the first state, which we named “cyan”, included all of the experimentally characterized interbands. Further, we identified three more chromatin states which differed in their protein ensembles. Blue chromatin is enriched in RNApolII, although not as high as cyan chromatin is. Notably, blue chromatin is not associated with CHRIZ. Next chromatin state, named magenta, is completely devoid of interband-specific proteins. As for the green-state chromatin, it differs from the above three states in having no obvious protein specificity; hence it is not considered here in detail and these data will be published elsewhere. To summarize, the whole body of chromatin turned out to be divided into 4 states that differed in associated proteins.

**Figure 2:**
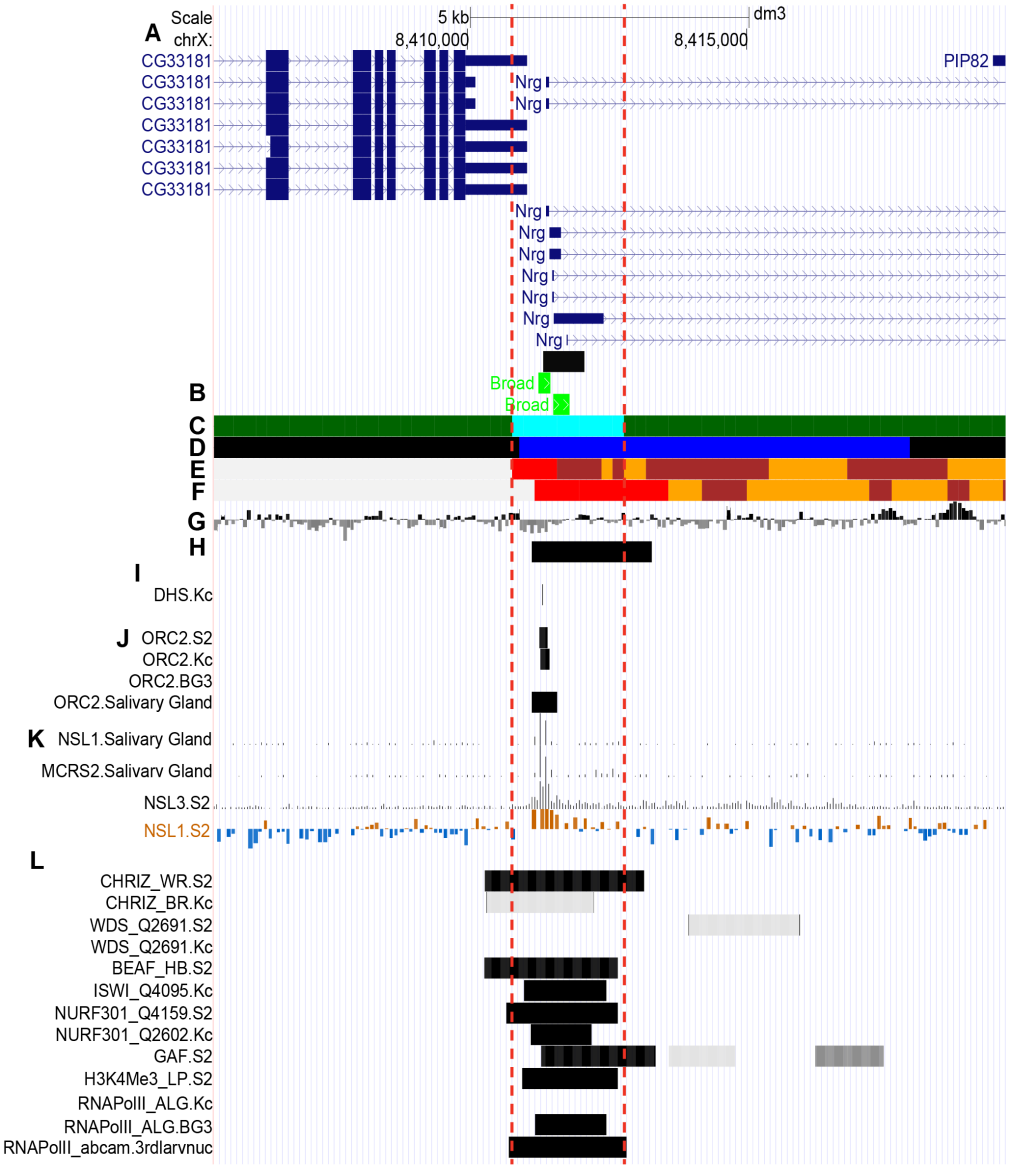
Localization of proteins and genomic features (fly modENCODE) in the interband 7F1-2/7F3-4. Vertical red dashed lines delimit the edges of cyan state chromatin in the region which conditionally reflects the location of the interband. **A –** gene map (RefSeq Genes). **B** – Localization of broad class promoters according to Hoskins et al. (2011) [42] (light green rectangles) and a FISH probe (black bar). **C** – Localization of 4-state chromatin types according to the algorithm developed in this paper. Only cyan and green chromatin types map to this genomic region. **D –** Five-state chromatin types in Kc cells by Filion et al. [20]. **E** - 9-chromatin states in S2 cells by Kharchenko et al. [22]. Chromatin state 1 is shown red. **F** - 9-chromatin states in BG3 cells by Kharchenko et al. [22]. Chromatin state 1 is shown red. **G –** Nucleosome density according to Henikoff et al. [43]. Peaks above the axis reflect high density and those below axis denote low nucleosome density. **H** – Localization of histone H1 dips in Kc cells by Braunschweig et al. [44]. Black horizontal bars indicate the genomic regions with low histone H1 binding. **I** - DNAse I hypersensitivity sites (high magnitude DHS - vertical lines) in S2, BG3, and Kc cells by Kharchenko et al. [22]. **J** - ORC2-binding sites in S2, BG3, Kc cells and salivary glands by Eaton et al. [45], Sher et al. [46]. **K –** Enrichment profiles of NSL complex components: NSL1 binding profile from salivary glands by Raja et al. [47], NSL3 in S2 cells by Lam et al. [48], NSL1 in S2 cells by Feller et al. [49]. **L –** Enrichment regions of various proteins specific for interbands and active chromatin (fly modENCODE). The list of interband-specific proteins is taken from Demakov et al. [28] and Vatolina et al. [27]. (See text for more detailed explanations). list of interband-specific proteins is taken from [27]–[28], [50]–[53]. (See text for more detailed explanations).

As is shown in Fig. 2, localization of proteins that largely define these states varies significantly between different experiments (for instance, compare CHRIZ WR.S2 and CHRIZ BR.KC profiles). Our mathematical pipeline processes the regions occupied by interband-enriched proteins so that their positions are averaged across the experiments and so the coordinates for localization region are produced that fit best all the individual enrichment profiles. The coordinates thus obtained define the borders of cyan chromatin state. Its span and coordinates on the physical map are used to conditionally define localization of DNA sequences that we attribute to interbands (Fig. 2, Figs. S3–S5).

The fraction of the *Drosophila* genome occupied by the chromatin states identified and the number of fragments are: cyan – 12.7% (5674 fragments), blue – 16.8% (4006 fragments), green – 22.5% (8903 fragments) and magenta – 48.0% (5148 fragments). The sizes of the cyan chromatin fragments range between 0.2 and 39.6 kb (2.7 kb average), blue - 0.2-46.8 (4.9 kb average), green - 0.2-46.8 kb (3.1 kb), magenta - 0.2-82.8 kb (11.2 kb average).

Genome browser-compatible tracks showing the positions of all four chromatin states can be found in the File S1.

#### Protein and genomic features in bands and interbands of polytene chromosomes

In our downstream analysis, we used a set of 32 interbands described above and whose positions on the cytology map were established with maximum accuracy. This set of interbands is also molecularly well-characterized. Therefore, it can be used for fine analysis of interbands, i.e. for mapping of proteins and functional chromatin elements enriched in these interbands. For each of these interbands, detailed maps of associated proteins and other open chromatin features were constructed; Fig. 2 provides an example of such maps.

Interband 7F1-2/7F3-4 shows nearly perfect overlap between the FISH signal from the 5′-*Nrg* probe, cyan state, various promoter types identified by Hoskins et al. [42], and active chromatin marks, such as CHRIZ, RNAPolII, nucleosome remodelers WDS, ISWI, NURF301, H1 dips, promoter-enriched H3K4me3 [50], DHS and red chromatin state (state 1 as defined by Kharchenko et al. [22]). Furthermore, of the entire 7F region only interbands display significant enrichment for ORC2 and NSL complexes.

Very similar trends are clearly observed for all other interbands as well (Figs. S3–S5): although protein profiles do show minor variability, overall most of the protein-enriched regions fall within the borders of cyan chromatin (red dashed line in Fig. 2, Figs. S3–S5).

**Figure 3:**
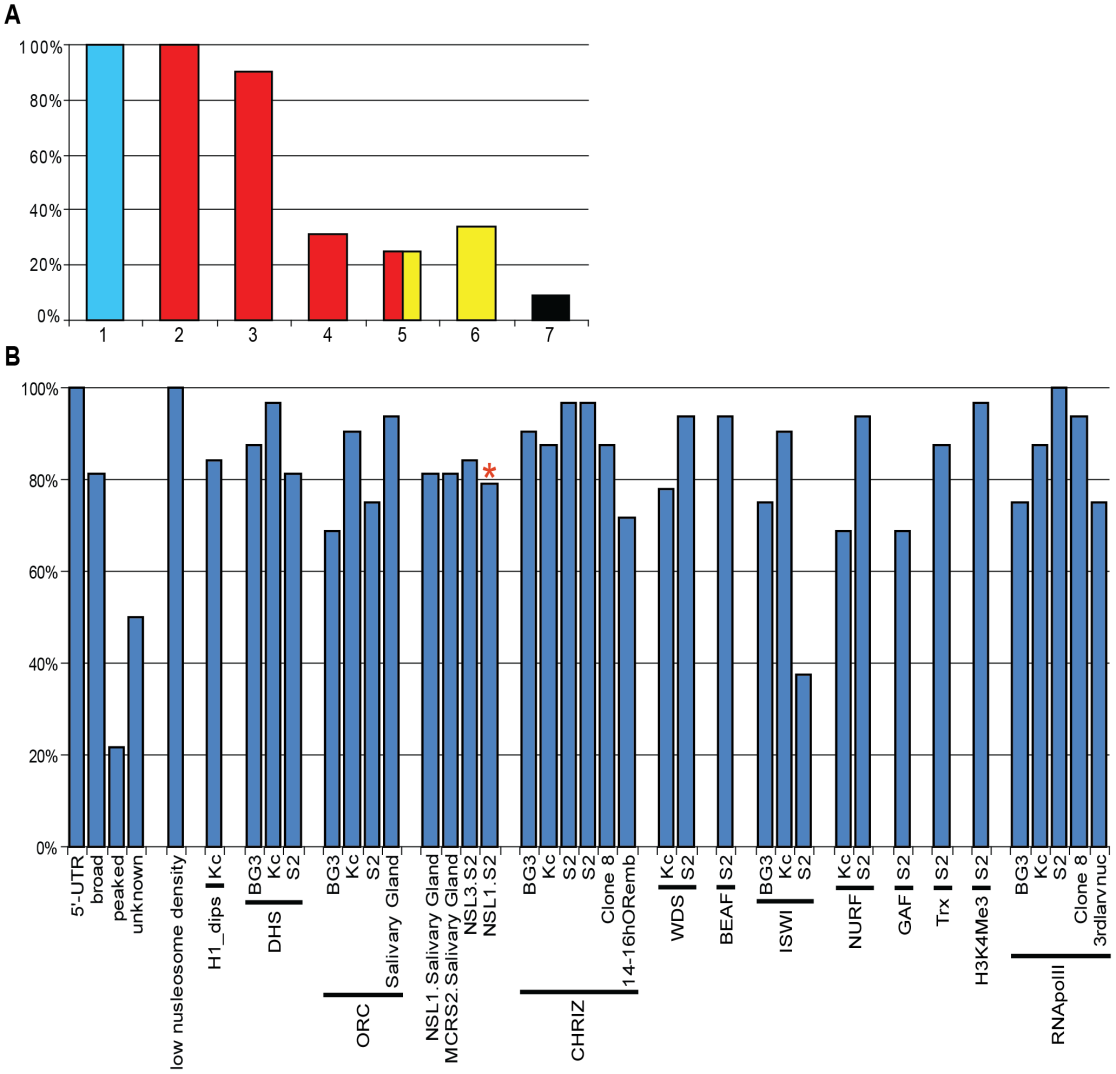
Positions of protein enriched regions, chromatin states and other genomic features in 32 cytologically defined interbands. A – Frequencies of chromatin states in select 32 interbands: cyan (1-this paper), state 1 (red chromatin) in 9 state chromatin model in BG3 (2) and state 1 (red chromatin) in S2 cells (3-according to [22], RED, YELLOW, RED/YELLOW and BLACK/BLUE chromatin types (4–6) according to Filion et al. [20]. B – Occurrence of various chromatin states, proteins and other genomic features in interbands. X axis - proteins or genomic features found in different cell cultures. Y axis shows a fraction of interbands demonstarting these characteristics. *Since some of the data were originally missing from the analysis, the frequencies are presented for a set of just 19 interbands.

All 32 interbands studied here display similar organization. First of all, 100% of interbands harbor cyan chromatin fragments, 5′-UTRs of genes, they show low nucleosome density and overlap with the positions of state 1 chromatin (red in 9-state model by Kharchenko et al. [22]) (Fig. 3A, B). Vast majority of interbands also display a number of features characteristic of transcriptionally active regions, namely broad-class promoters, H1 dips, DHS, RNApolII (Fig. 3), CHRIZ and BEAF-32. In all the cytologically defined interbands, we observed enrichment for replication complex components (ORC2) (Fig. 3).

Our set of interbands displays clear enrichment for NSL complex proteins, which have been reported to specifically associate with promoters of multiply active genes [48], [49]. If one compares NSL localization data from *Drosophila* salivary gland polytene chromosomes with the interband-mapping data, the overlap is nearly perfect (see Fig. 3B).

What would be the controls for whether the interband fragments have been correctly identified and whether the whole idea of mapping the interbands on a physical map is robust? We believe one of the solutions would be to do a reverse experiment, i.e. to perform polytene chromosome mapping of transposons that we determined to have landed into cyan chromatin fragments.

We selected three matching transposon lines: w* P{EP}G400, *y^1^ w^67c23^ P{EPgy2}Hsp60^EY01572^* and *y^1^ P{EPgy2}EY09320 w^67c23^* located in cyan regions. FISH analysis of polytene chromosomes from these transgenic stocks using *white* DNA as a probe (as all these transposons are *white*-marked) shows that for w* P{EP}G400 the FISH signal maps immediately proximal to 10A1-2, i.e. it is located in the adjacent interband (Figure S6A, D, G). The insertion of *P{EPgy2}Hsp60^EY01572^* lies a little proximal, in the interband 10A3/10A4-5. Accordingly, FISH signal is also found more proximal, i.e. in the polytene chromosome interband 10A3/10A4-5. In this case, there is a small gap between the band 10A1-2 and the FISH signal (Figure S6B, E, H). Insertion of *P{EPgy2}EY09320 w^67c23^* maps to the interband 10A7/10A8-9 of a physical map, which is consistent with the FISH signal localization in the same polytene chromosome interband (Figure S6 C, F, I).

**Figure 4:**
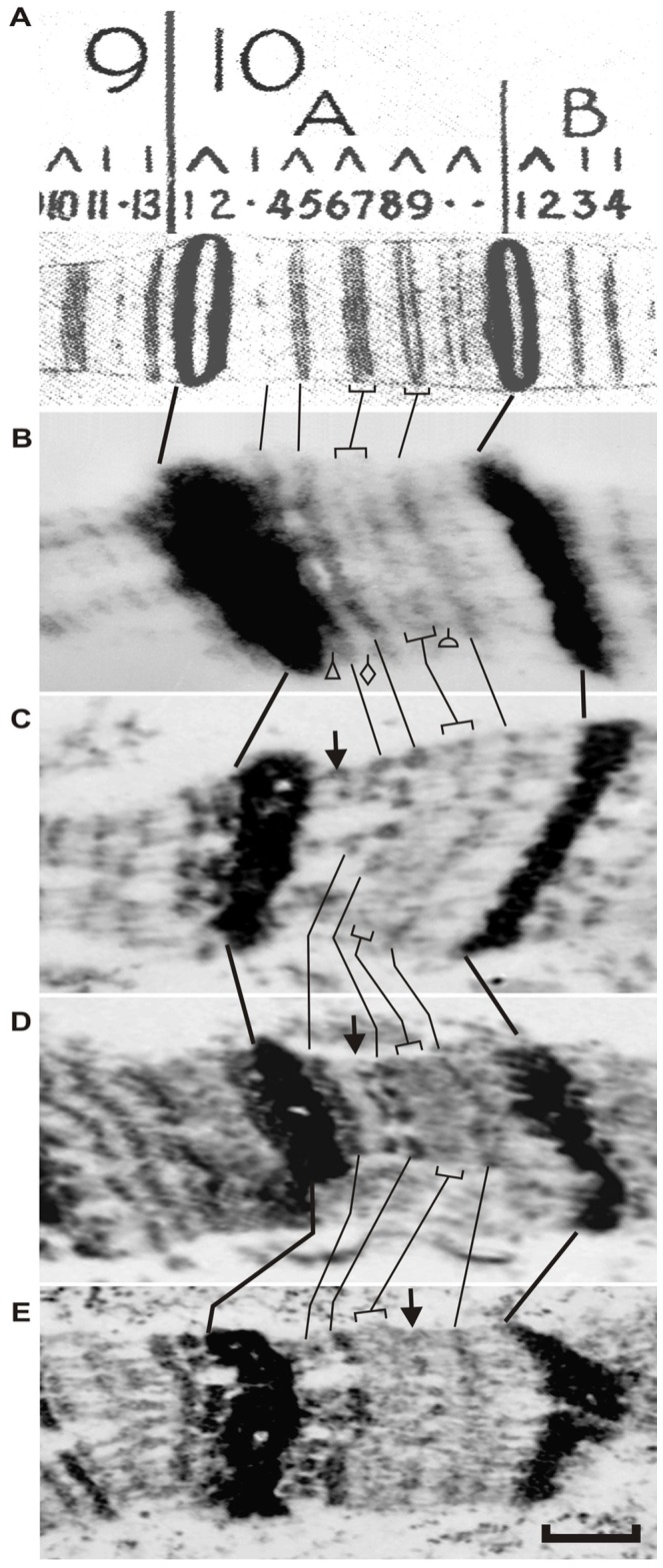
EM mapping of transposon insertions in the predicted interbands, 10A1-2/10A3 (C), 10A3/10A4-5 (D), and 10A7/10A8-9 (E) in salivary gland polytene chromosome X. A – Fragment of the Bridges' map of the X chromosome [35]; B – Electron micrograph showing morphology of the region 10A in wild-type X chromosome; C - E – polytene chromosomes in *Drosophila* lines containing w* P{EP}G400, y^1^w^67c23^P{EPgy2}Hsp60^EY01572^ and y^1^P{EPgy2}EY09320w^67c23^ transposons, respectively. Location of the insertion site of P{EP}G400 transposon on the chromosome map of wild type is indicated by triangle in wild type chromosome (B) and by an arrow in the insertion line (C). For insertion y^1^w^67c23^P{EPgy2}Hsp60^EY01572^, the integration site is shown as diamond (B) and arrow (D). The same for insertion of y^1^P{EPgy2}EY09320w^67c23^: semicircle (B) and arrow (E). Bar corresponds to 1.5 mkm.

Using EM analysis of these regions, we found three novel bands in exactly the interband regions we expected (arrows in Fig. 4C-E). This serves as independent and very important evidence arguing in favor of correct identification of interbands, based on the protein localization data in the interbands from the 9F13 to 10B3 region. It must be emphasized that mapping of interbands followed by transposon localization stemmed from protein localization data in interphase chromosomes of mitotically active cells. This allowed tracking the transposon insertions into these same interbands, yet in the context of polytene chromosomes.

**Figure 5:**
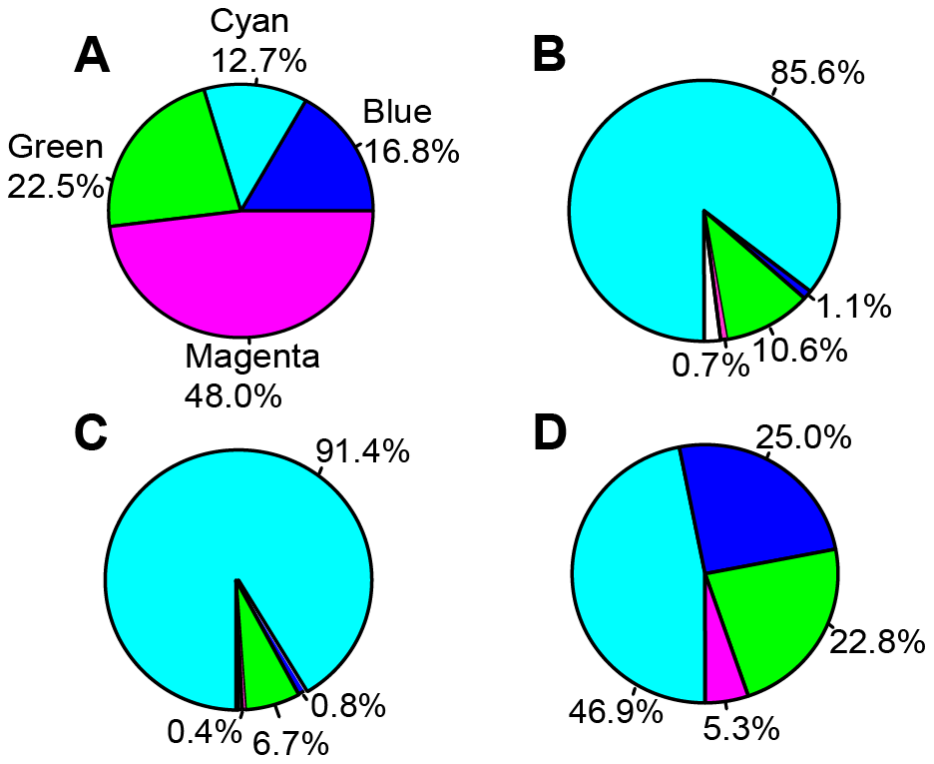
Pie charts showing proportions of four chromatin types in the genome (A), genome-wide distribution of DHS (B), ORC2 sites (C) and histone H1 dips (D) in these chromatin types in the *Drosophila melanogaster* genome (Kc cells). Chromatin types are color-coded according to their names: cyan, blue, magenta, green. Unshaded sectors denote absence of the data in modENCODE: 2% (B) and 0.7% (C).

These data are consistent with the idea that cyan chromatin fragments correspond to interband DNA, moreover the chromatin features such as ORC2 and DHS preferentially map to interbands as well. Therefore, it is straightforward to analyze the distribution of chromatin features such as ORC2, H1 dips and DHS (the features that were not used to compile the 4-state model) across the genome. Results of this genome-wide analysis are presented in Fig. 5 and show that in Kc cells 85.6% DHS, 91.4% ORC2 and 46.9% sites of histone H1 dips overlap with cyan chromatin. Similar numbers are observed for other cell lines (S2 and BG3) as well as for larval salivary glands (data not shown).

#### Genes vs bands and interbands

**Figure 6:**
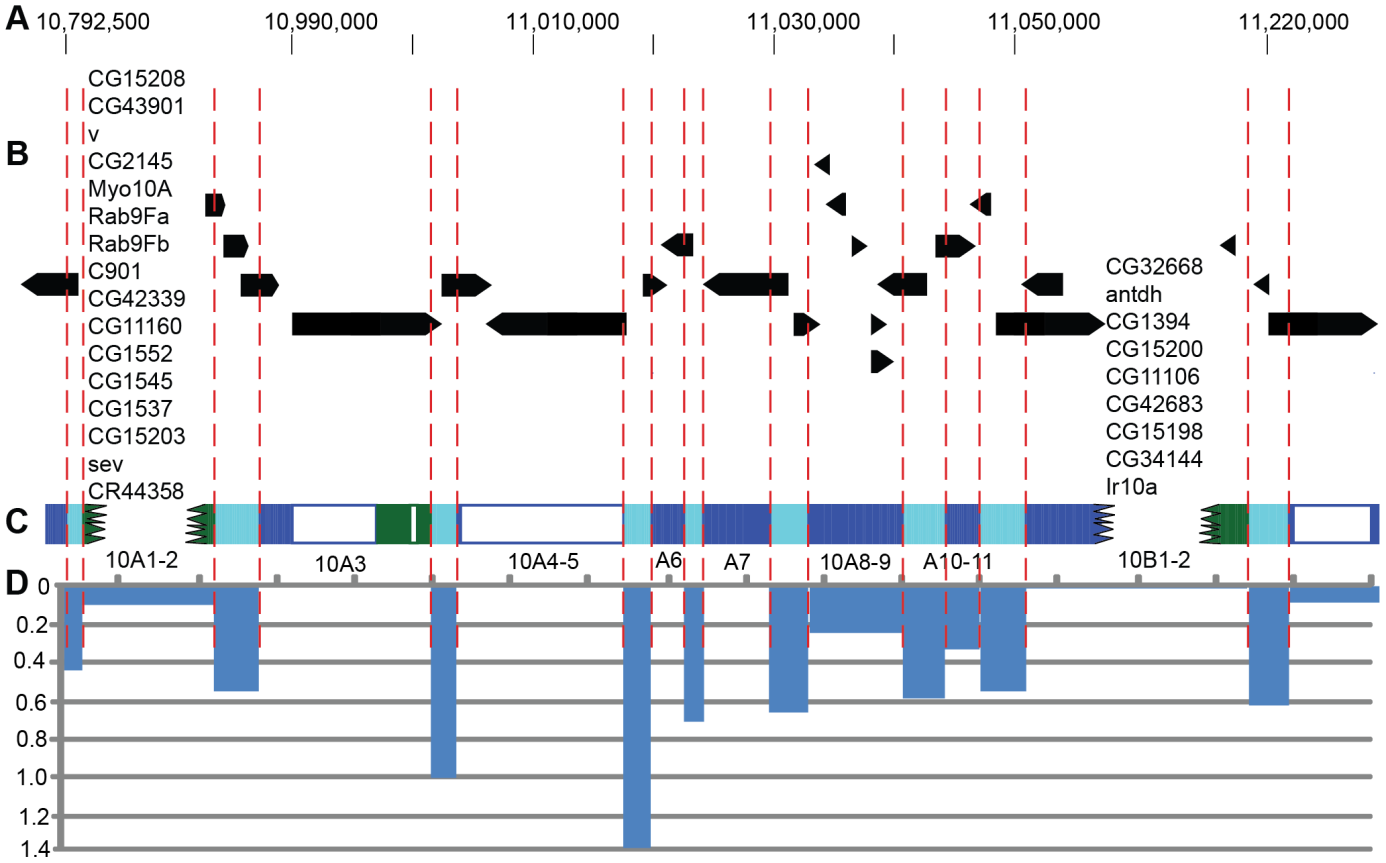
Comparison of the banding pattern and gene positions in the region 9F13 – 10B3 of the X chromosome drawn to the same scale. A – Genomic coordinates. B – positions of genes (RefSeq genes) on the physical map. Genes in the bands 10A1-2 and 10B1-2 are listed randomly and the extents of these bands are depicted as a jagged line. C – location of four-state chromatin types described in this study. Vertical red dashed lines delimit the borders of bands (labeled below) and interbands according to the borders of cyan fragments. Unshaded areas reflect absence of the data in modENCODE. D – frequencies of P-element transposon insertions (per 1 kb) averaged across the span of a band or an interband.

As was mentioned above, in polytene chromosomes there are three basic structural types: interbands and two types of bands – large IH bands composed of tightly compacted late-replicating material, and loosely compacted small bands replicating early. High-resolution mapping of chromosomal structures presented in this study makes it possible to match the positions of genes and chromosomal structures on a scale of the physical map. One such comparison for the region 10A is illustrated in Fig. 6.

In polytene chromosomes, two prominent late-replicating IH bands 10A1-2 and 10B1-2 flank a group of six faint loosely compacted bands that are barely detectable under the light microscope. They are depicted grey on Bridges' map [35], and look similarly grey on the EM map (see Fig. 4). Thus, this region encompasses 7 interbands. The schematic figure of this genomic region clearly shows 6 alternating pairs of cyan/blue chromatin states (Fig. 6), much like the number of bands and interbands. In genetic terms, cyan chromatin corresponds to the 5′-regulatory part of the gene (alternatively, small genes are entirely engulfed by cyan chromatin). Given that cyan chromatin is a defining feature of interbands, then blue chromatin can only map to the space between two interbands, i.e. to the neighboring loosely compacted grey bands. On the molecular map, these interleaving blue and cyan chromatin types perfectly mirror the pattern of alternating bands and interbands on the cytology map (Fig. 6).

Yet another region, 100B, may serve to further illustrate of this trend. As was mentioned above, this region harbors a group of two interbands and a loosely compacted very faint band 100B3 (Figs. S1) barely visible under EM. These structures are flanked by late-replicating bands 100B1-2 and 100B4-5 [34] (green fragments on Fig. S3). The two interbands distal and proximal to 100B3 display all the features characteristic of interbands. On a physical map, the coding sequence of *dco* gene of about 3 kb maps between these two interbands, hence it likely corresponds to the miniature band 100B3. Thus, in the regions where interbands alternate with grey bands, the interbands generally tend to comprise 5′-ends of the genes and correspond to cyan chromatin state, whereas grey bands harbor gene coding sequences. This observation forms the basis of a hypothesis that in the context of interphase chromosome, many other regions may share the same pattern of genetic organization (interband – 5′-end, loose grey band – gene coding sequence).

So, the genes mapping to grey bands/interbands, in fact, may occupy two polytene chromosome structures: interband hosts the 5′-end of a gene encompassing its regulatory part, the first exon and intron, whereas the structural part of the gene is found in the neighboring loosely compacted grey band. In this respect, localization of cyan fragments serves as a marker of 5′-ends of genes and interbands, while blue chromatin may correspond to loosely compacted grey bands and coding parts of the genes. Thus, loosely compacted grey bands and the adjoining interbands can be viewed as linked structures: gene promoter locates to the interband, gene coding region resides in the adjacent grey band.

In the set of 32 interband/grey bands studied here, we identified 65 genes (Table S1). Late-replicating IH bands are dramatically different from the loosely compacted grey bands in that they comprise densely packed chromatin: they replicate late, they lack typical interband- or grey band-specific proteins, and instead associate with SUUR, D1, lamin B, histone H1 and other proteins characteristic for BLACK chromatin [20], [23], [33]. 238 genes were found in the late-replicating IH bands 7F1-2, 7F3-4, 10A1-2, 10B1-2, 19E1-4, 21D1-2, 21E1-2, 35D1-4, 56A1-5, 58A-B1-2, 70A1-5 and 100B1-2 – 100B4-5 located next to the 12 reference interbands (Table S1).

#### Preferential integration of P-elements in interbands as a feature of open chromatin

Our earlier analysis of 12 interband regions from polytene chromosomes [32] reported that in the *Drosophila* genome, P-elements preferentially integrate into interbands. Genome-wide analysis was indicative of predominant integration of P-element transposons into replication origins (ORC-positive regions) [54], which in turn tend to largely locate to interbands [26], [27].

We further confirm this observation, as we compare the insertions of transposons and chromosomal structures. Clearly, the interbands serve as the hotspots of transposon insertions, which is evident on the molecular and genetic maps of all interbands studied to this end (Fig. 6D).

**Figure 7:**
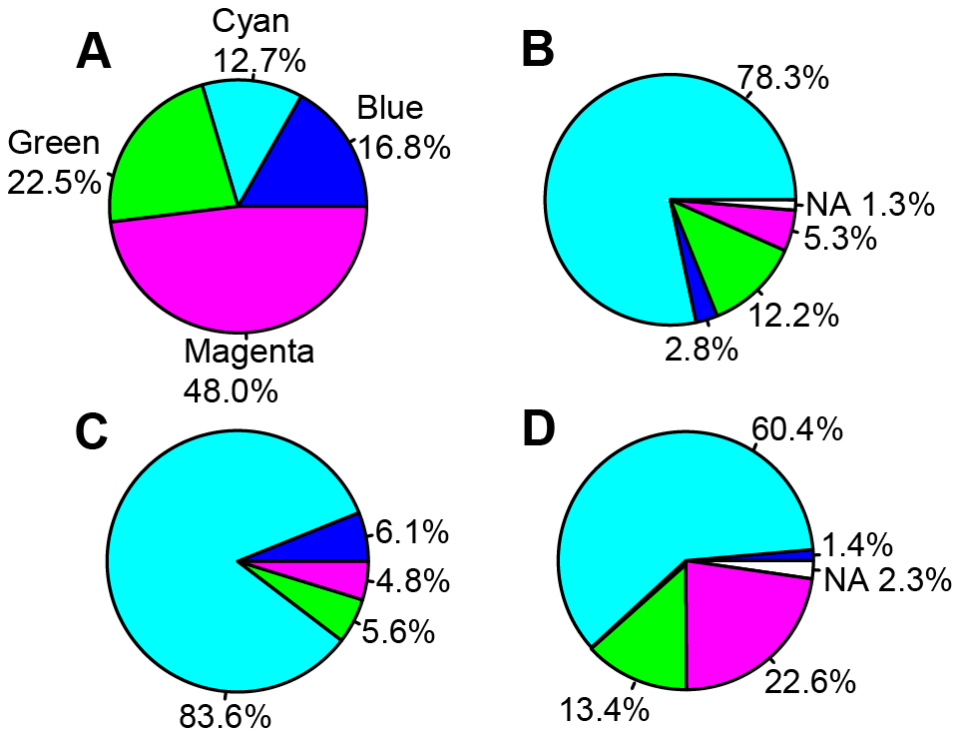
Pie charts showing frequencies of various genomic features mapping to the different four-state chromatin types (A): P-element insertions (B), localization of "broad" type promoters (C) and “head-to-head” arrangement of genes (D) in the *Drosophila melanogaster* genome. (see text for further explanations).

When this analysis is performed genome-wide, and localization data for all the P-element insertions referenced in the FlyBase (38,888 insertions) are used, 78.3% of all insertions map to the cyan chromatin states, which constitutes only 12.7% of the genome sequence (see above). This translates into 6.2-fold higher frequency compared to the random distribution of insertions. Importantly, we observe a pronounced decrease in insertion frequencies in other chromatin types. P-element based transposons are 9.1-fold less likely to land in magenta chromatin (6.0 and 2.0-fold for blue and green, respectively (Fig. 7B) (X = 108327.3, p value <2.2e-15, Mann-Whitney test).

P-elements are known to typically transpose in diploid germline cells. On the other hand, we and others have observed that P-element transposons tend to insert into open chromatin regions [32]. Consequently, one may speculate that regions appearing as interbands in the context of polytene chromosomes should be similarly found in an “open chromatin” state in the germline.

#### Interbands correspond to 5′ regions of ubiquitously active genes

Distinct chromatin organization observed for genes residing in three basic types of polytene chromosome structures selected for this analysis (IH bands, interbands and loosely compacted grey bands) is suggestive of their specific organization and function. We compared expression patterns of genes found in the late-replicating IH bands vs those located in 32 interbands/grey bands (see the list in Table S1). Quantitation of gene expression across 8 larval and 17 adult organs has been reported in Chintapalli et al. (FlyAtlas) [55]. We observed that gene activity varied depending on which structure the gene mapped to.

**Figure 8:**
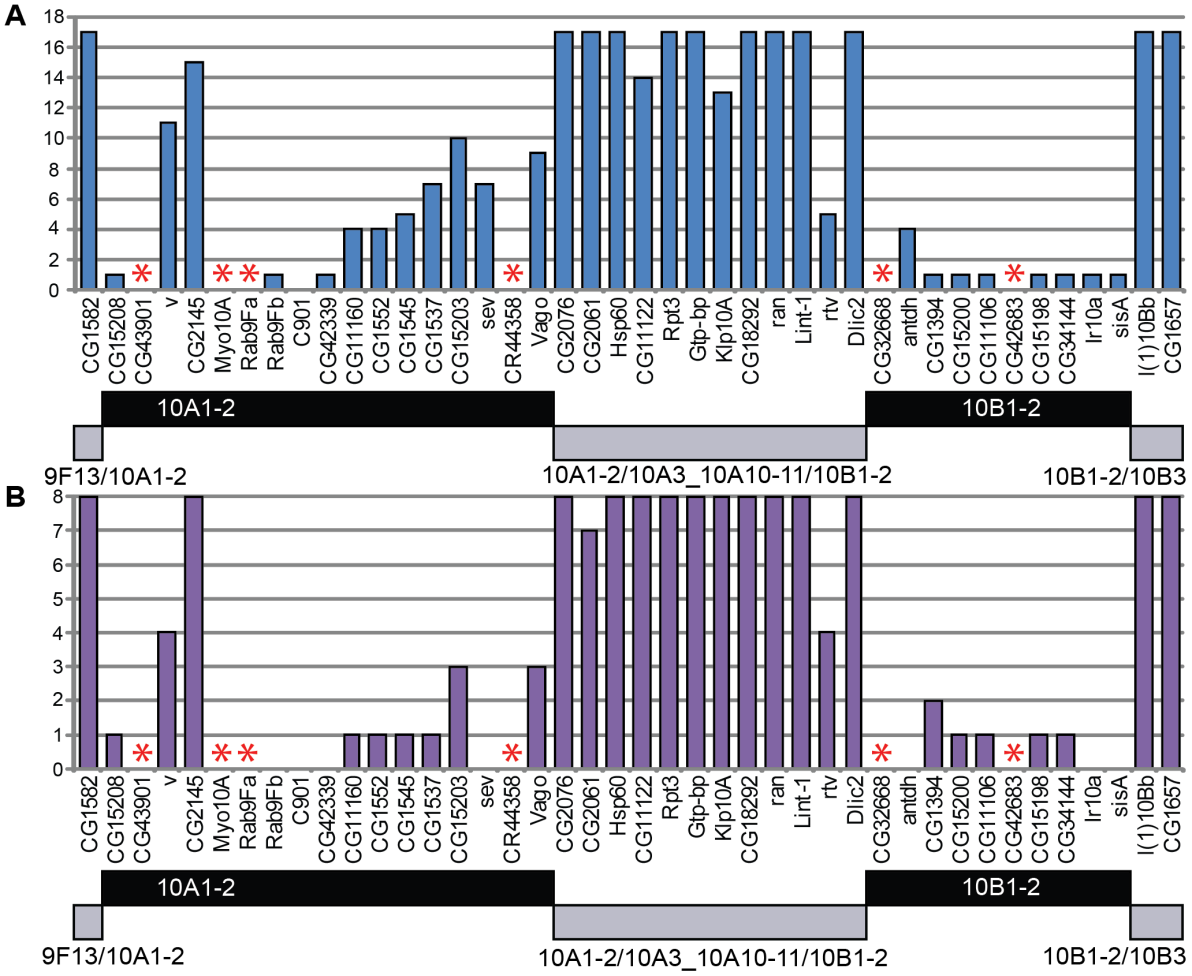
Activity of “band” and “interband” genes located in 9F13 – 10B3 region. Genes are listed below the *x* axis. The number of tissues where gene activity was found in adult flies (totally 17 tissues studied) (**A**) and larvae (totally 8 tissues studied) (**B**) is plotted on the *y* axis. Data on gene activity were taken from Chintapally et al. [55]. Horizontal bars below the *x* axis denote the extent of the bands 10A1-2 and 10B1-2 (black) and alternating interband/grey bands (grey), as well as two interbands (grey) on the very edges of the region. Asterisks – NO data.

**Figure 9:**
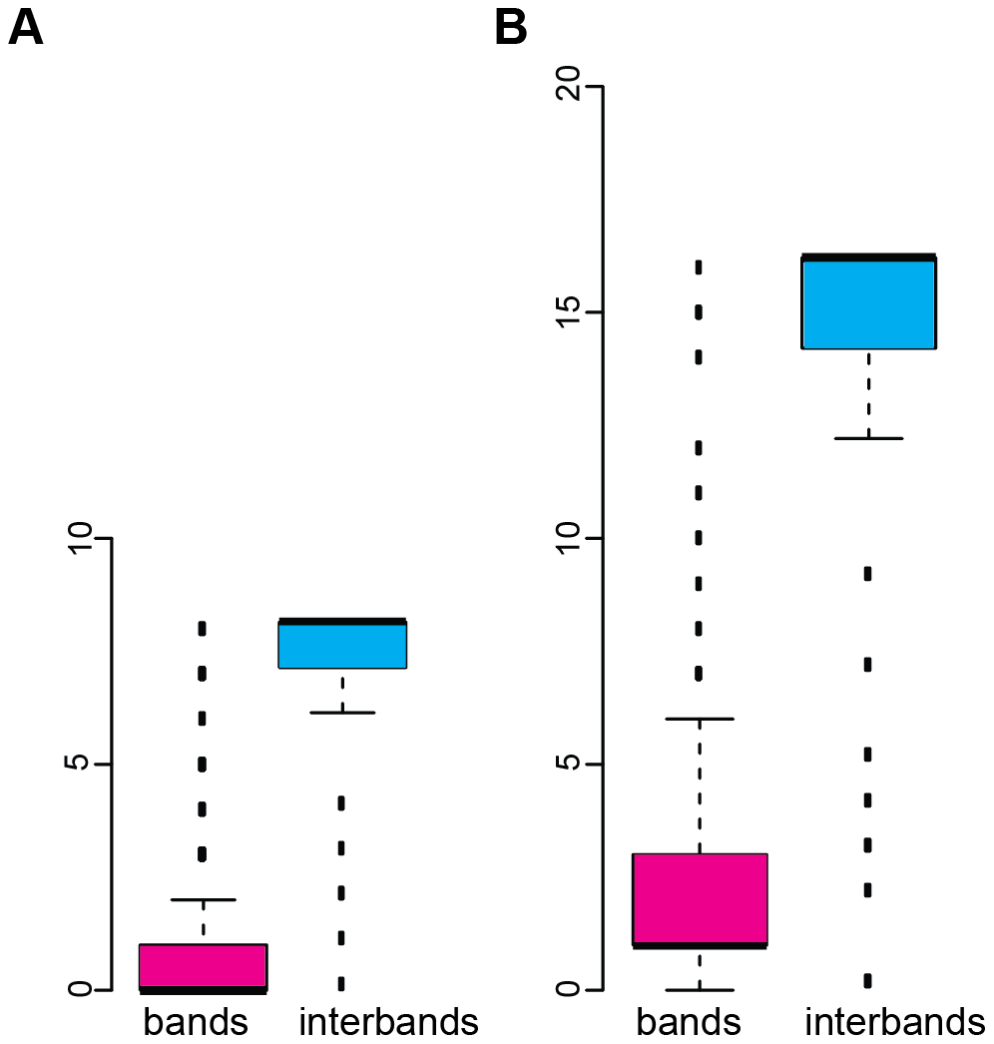
Box-and-whiskers plot showing mean number of larval and imaginal tissues where the activity of “band” and “interband” genes was found. Y axis: number of tissues profiled in larvae (A) and imago (B). Median positions are shown as thick black bars. Error bars (blue and red boxes) represent the data between 25 and 75 percentile (50% of data points). Dataset range between the largest and the smallest values is shown as thin horizontal bars. Dashed line represents outliers. To construct this plot, data from Chintapally et al. [55] were used.

In the region 9F13 – 10B3, interband/grey band genes (genes within the interband 9F13/10B1-2, all genes between 10A1-2 and 10B1-2, and in interband 10B1-2/10B3) are active across almost all tissues analyzed. At the same time, expression pattern of genes found in large IH bands 10A1-2 and 10B1-2 is much more restricted (Fig. 8). Similar trends were also observed when comparing gene activity in other interband/grey bands vs IH bands mapped in this study (Fig. 9). On average, the transcripts from genes that overlap with the studied interbands are likelier to be present in more tissues, as compared to the transcripts mapping to IH bands, - both in larvae and adults (p-value ≤2.2e^−16^ Mann-Whitney test).

To compare the magnitude of transcription for the genes found in IH bands and 32 interbands (see the list of genes in Table S1), we calculated average expression levels for each gene present on the microarray (FlyAtlas Anatomical Expression Data) [55]. We estimate that interband-resident genes have 27-fold higher median expression (112 vs. 4.2) than those mapping to IH bands (Figure S7).

Next, we performed a more comprehensive genome-wide analysis of gene activity in cyan and magenta chromatin states. On average, median expression levels for genes in cyan fragments was 20.9 times higher than those in magenta chromatin (100.4 vs 4.8) (Figure S8).

Recently, many interesting datasets characterizing *Drosophila* transcriptome have been published, utilizing high-throughput RNA-seq technique (Gelbart, W.M., Emmert, D.B., 2013 FlyBase High Throughput Expression Pattern Data). For each gene, abundance of RNA transcripts was measured throughout development (whole organism, 30 developmental stages) [55], in different organs (29 dissected tissues) (modENCODE Tissue Expression Data), in cell lines of various origin (24 cell lines) (modENCODE Cell Line Expression Data), and upon different experimental treatments (21 treatments of whole animals with various toxins) (modENCODE Treatment Expression Data) (http://www.ncbi.nlm.nih.gov/pmc/articles/PMC3032933/).

Using these data, we confirmed that expression of 238 genes mapping to IH bands (see the list in the Table S1) is significantly lower (median expression is almost zero) than expression levels observed for 32 interband genes (median expression is 15 RPKM) (Figures S9–S10).

On a genome-wide scale, we observed maximum activity of genes whose 5′-ends mapped to cyan chromatin fragments (median expression values are around 11) in comparison with magenta fragments (median expression values are close to 0) (Figures S11–S12).

Taken together, these data are indicative of the constantly high transcription level of genes whose 5′-ends locate to the interbands or to the cyan chromatin state in the genome. These genes are active across the majority of cell types (i.e. they may be referred to as ubiquitously active or multiply active genes).

#### Promoter architecture in genes residing in interbands and bands

Recent high-resolution mapping of promoters active in the *D. melanogaster* embryos identified 12454 promoters in 8037 genes. It was shown that distribution of transcription start sites (TSS) within these promoters forms a complex continuum of shapes, and that promoters active in the embryo and adult have highly similar shapes in 95% cases. This led to the conclusion that these distributions are generally determined by static elements such as local DNA sequence, rather than by dynamic signals such as histone modifications. As it turned out, *Drosophila* promoters are characterized either by broad region of distributed TSSs, or by single TSS defining a discrete promoter. These patterns are consistent with a definition of “broad” and “peaked” promoter classes in the human and mouse genomes [42]. Peaked promoter shape is correlated with both temporal and spatial regulation of gene expression [42]. Of the 32 interband regions studied in the present paper, 26 encompass gene promoters of various architectures, yet nearly invariably including broad class promoters (81.2% of interbands); 6 interbands lack broad promoters, as they host either unknown or peaked promoter classes (18.8%). Thus, interbands generally include broad promoters.

On a genome-wide scale, 83.6% (6436) of broad-class promoters map to cyan (interband-enriched) chromatin fragments (Fig. 7C). This argues that broad promoters predominantly map to interband regions.

Interestingly, analysis of promoter architecture in the human and *Drosophila* genome showed high frequency of bi-directional promoters activating expression of genes arranged in a “head-to-head” configuration with less than 2000 base pairs of intervening sequence [57], [58]. It is possible that such gene orientation contributes to enhancing gene transcription activation and elongation.

Of the genes located in 32 interbands listed in Table S1, bi-directional promoter architecture was observed in 13 cases (41% interbands). In the *Drosophila* genome, there are 3357 head-to-head oriented genes with less than 2000 base pairs between them (FlyBase). Of these, 5′-ends of 2027 genes (60.4%) map to cyan chromatin, which constitutes only 12.7% of the genome. In stark contrast, only 46 “bi-directional” genes (1.4%) are found within blue chromatin (16.8% genome). Green (22.5%) and magenta chromatin types (48.0%) encompass 448 (13.4%) and 760 “bi-directional” genes (22.6%), accordingly (Fig. 7D). Clearly, interbands are enriched for this particular class of genes in a head-to-head orientation, whereas all other chromatin types are significantly depleted for this feature (X-squared = 1208.28, p-value<2.2e-16).

## Discussion

The present study aims at unraveling the genetic and functional organization of basic morphological features of interphase chromosomes. In the context of polytene chromosomes, these features display distinct degrees of chromatin packaging and comprise interbands, loosely compacted grey bands and dense IH bands. We attempted to correlate positions of gene elements, gene expression and the epigenetic state of underlying chromatin for these structures. To do so, we first had to accurately locate these morphological elements on the physical map of the genome. This allowed us to compare their positions with genetic and epigenetic maps, as well as with protein localization profiles, transcription profiles and other features of chromatin. So, we could relate functional domains with the banding pattern of polytene chromosomes.

Dense black bands are the most prominent structures in polytene chromosomes. They are readily noticeable due to their highly compacted state, large size, lack of transcription, late replication in the S phase, and a tendency to form ectopic pairing with other bands and pericentric heterochromatin. In fact, black bands are in many regards very similar to pericentric heterochromatin, hence they were called IH [59], [60]. In polytene chromosomes, IH bands frequently fail to complete replication during the S phase endocycles, and are therefore underreplicated. It has recently become clear that underreplication results from the absence of internal replication origins within IH and is dependent on SUUR protein, which maps to IH bands and modulates replication [34], [61] by decreasing the rate of replication fork progression [46].

Underreplication regions showing lowered DNA copy number in polytene chromosomes were molecularly mapped [25], [61], [62]. This analysis established that IH bands encompass clusters of widely-spaced unique genes (i.e. genes with large intergenic regions, with 6–40 genes per IH band), and that they are generally quite large (100–600 kb) [24], [25]. Combined with the data on localization of chromatin proteins [20], [22], IH borders were precisely mapped for 60 IH regions, which enabled a more refined analysis of these structures [26].

Our data (present paper) and those of Filion et al. [20] indicate that IH bands are composed of tissue-specific genes showing low expression levels. One of the prominent features of IH regions is their evolutionary conservation, i.e. they tend to display conserved gene content and order throughout evolution, as has been demonstrated by microsynteny analysis in nine *Drosophila* species [63].

As compared to IH bands, it is far less trivial to provide accurate mapping for interbands and grey bands, because these regions are fully replicated and are much smaller. Yet, using a combination of EM, P-element tagging and FISH, we were able to unambiguously map the positions of 32 interbands. Using the data on the features of interband chromatin, we developed a mathematical model that defines four basic chromatin states in the drosophila genome. This model allows identification of interband regions chromosome-wide. Accordingly, the limits of the DNA sequences corresponding to interbands were defined as borders of cyan fragments.

With these data in hands, we proceeded to analyze the molecular and epigenetic organization of interbands. Interbands clearly displayed features of transcriptionally active regions: H3K4me3 histone modification, lower nucleosome density and histone H1 dips, presence of DHS, localization of RNA polymerase II and components of nucleosome remodeling complexes such as NURF, ISWI, WDS. One characteristic feature of interbands is that they are specifically bound by the chromodomain-containing CHRIZ protein [41], [51], [52], [64]. CHRIZ associates with another interband-specific protein Z4, which directly binds DNA via its seven zinc fingers [52], [65].

According to different estimates, there are 3500–5000 bands and interbands in *Drosophila melanogaster* polytene chromosomes [18]. Earlier, we predicted the existence of about 3500 interbands [28]. Here, we use an advanced model that takes into account more factors and hence is more accurate. We found 5674 cyan fragments each spanning 2.7 kb on average. Notably, both our previous and current estimates of interband numbers are very close to those obtained by cytology.

The major finding of our analysis of functional organization of interbands is that they typically encompass 5′-regions of multiply active genes (constitutively and actively transcribed). In a number of instances, we observe that short genes can be entirely engulfed by interbands (see Fig. 5), however in most cases the body of the gene is found in the adjacent loosely compacted grey band. Thus, the interband+grey band duo appears as a single functional unit for many multiply active genes, so this unit is heterogeneous in terms of compaction; likewise it shows non-uniform localization of protein markers. Whereas interbands are specifically decorated with CHRIZ, grey bands lack CHRIZ and instead they are enriched with RNApolII. CHRIZ can be speculated to provide the permanently open chromatin state to interbands, where it serves as a pioneer-factor recruiting other transcription components. It is also possible that the observed wide-spread transcription activity of interband regions results from the static physical properties of interband DNA, such as sequence-dependent DNA flexibility, which may create nucleosome-free regions at promoters. Such regions may serve as “entry points” to recruit proteins promoting further binding of transcription factors, chromatin remodelers, etc [66].

Our findings, therefore, resonate well with several early ideas regarding the interplay of structural and functional organization of banding pattern in polytene chromosomes. These include interband localization of multiply active genes, and the hypothesis of a single functional unit composed of band+interband (see Introduction).

Recently, there has been an avalanche of publications describing various types of domain organization in the genomes of eukaryotes [20], [22], [67], [68]. So, our data can be conveniently compared with other genome-wide chromatin annotation projects. Figure S13 summarizes domain organization of a 400 kb fragment of the X chromosome encompassing various types of bands and accurately mapped interbands. This figure shows that two large domains, 189 and 170 kb long, correspond to polytene chromosome bands 10A1-2 and 10B1-2, and display features of intercalary heterochromatin (magenta-green chromatin states) (Figure S13 A-B). In between these late-replicating domains, there is a region composed of alternating interbands and grey bands (cyan and blue fragments) (Figure S13 B). When applied to this region, 5-state chromatin classification model by Filion et al. [20] produces very similar domains, - the important difference however is that regions of YELLOW chromatin (active gene transcription according to Filion et al. [20]) do not discriminate between small grey bands and interbands, nor between regulatory vs gene body parts (Figure S13 C).

Kharchenko et al. [22] performed genome-wide profiling of 18 histone modifications and constructed 9-state chromatin models. As is shown in Figure S13 D, E, in two contrasting cell lines (S2 and BG3), transcriptionally silent chromatin corresponds to the IH bands 10A1-2 and 10B1-2, whereas state 1 chromatin (active promoters and TSS) (shown red in the Figure S13 D, E) maps to the active genes and perfectly matches the cyan state of interbands, as defined by our analysis.

Using the modified Hi-C approach based on the ligation of chromatin fragments that are found in close proximity in cross-linked chromatin, high-resolution chromosomal contact maps were generated (reviewed in [21]). As it follows from this analysis, the entire genome is partitioned into a series of physical domains containing active and repressive epigenetic marks. These domains are delimited by boundaries demonstrating insulator binding, high DNaseI sensitivity and a set of specific proteins: CHRIZ and the active histone mark H3K4me3 [67], [68]. The regions of interbands match well with the boundary sites that delimit the contacting domains identified via Hi-C (Figure S13 F, G). We compared localization of cyan chromatin and physical domains throughout the genome. Of 1100 boundary sites referenced in [67], 760 (69%) map to interbands (cyan) and loosely compacted grey bands (blue chromatin).

Positions of interbands and loosely compacted grey bands display co-localization with clusters of multiply active genes [49], [69] (Figure S13 H, I). According to our analysis, there are 12 genes nested between the 10A1-2 and 10B1-2, with their 5′-ends mapping to cyan chromatin. Of these, 5 genes were classified as housekeeping genes in Weber and Hurst [69].

Using the data from Chintapalli et al. [55], Feller et al. [49] also classified the genes as “housekeeping” or “differentially regulated” (Figure S13 I). Under this classification, the region 10A1-2 – 10B1-2 harbors 11 housekeeping genes, of which 9 genes correspond to our definition of a multiply active gene.

NSL complexes are reportedly regulators of multiply active genes and bind promoters demonstrating broad transcriptional pattern and nucleosome-free regions [48]. Positions of NSL-binding peaks match nicely the interband positions found in our study (Figure S13 K-M). All these comparisons further confirm the main conclusion of our work about interbands as sites of continuously active genes.

## Materials and Methods

### *Drosophila* stocks

*Drosophila* stocks containing insertions of transposons w* P{EP}G400, *y^1^ w^67c23^ P{EPgy2}Hsp60^EY01572^* and *y^1^ P{EPgy2}EY09320 w^67c23^* were used. The stocks were kindly provided by the Bloomington Drosophila Stock Center. Flies were raised on standard cornmeal–yeast–agar–molasses medium [70].

### Electron Microscopy

Salivary gland polytene chromosome squashes were prepared for electron microscopy analysis and examined as described earlier [31], [70]. The 120–150 nm sections were cut using an LKB-IV (Sweden) ultratome and examined under a JEM-100C (JEOL, Japan) electron microscope at 80 kV.

### Fluorescence in situ hybridization (FISH)

Salivary glands were dissected in Ephrussi-Beadle solution, and then fixed in a 3∶1 mixture of ethanol and acetic acid for 30 minutes at −20°C, squashed in 45% acetic acid, snap-frozen in liquid nitrogen and stored in 70% ethanol at -20°C. Fluorescence in situ hybridization (FISH) on polytene chromosomes was performed as described [71]. Random-primed labeling of DNA probes with biotin-16-dUTP or digoxigenin-11-dUTP (Roche) was done using Klenow enzyme. All the probes used in this study are described in the Table S2.

### Immunostaining of polytene chromosomes

Immunostaining was performed as described [41], [52], [72]. For CHRIZ detection, primary rabbit polyclonal anti-CHRIZ antibody (1∶600 dilution) and secondary FITC-labeled goat anti-rabbit IgG-specific conjugates (Abcam, 1∶200) were used.

### Genome-wide analysis of P-element insertion sites

To analyze the distribution of P-element insertions within the euchromatic part of the genome (X, 2L, 2R, 3R and 3L arms, as defined in FlyBase), we used insertion coordinates tagged as “transposable_element_insertion_site” from FlyBase release 5.50. We had to exclude 718 insertions (1.85% of the total sampling) from further analysis, as they mapped to multiple chromatin types or to the regions where the model failed to return a specific value.

### Analysis of promoter shape

Using R-language, we quantitatively described the distribution of promoter shapes [42] across chromatin types predicted by our model.

### Analysis of gene orientation

To analyze promoter orientation in pairs of adjacent genes, we used gene models from FlyBase release 5.50. Gene pairs were chosen so that no annotated gene bodies mapped between these genes. A custom R script (available upon request) was used to analyze the overlap of gene pairs with chromatin states in our model. We assumed that the chances of two genes being oriented “head-to-head”, “tail-to-tail” and “head-to-tail” under random distribution would be 25%, 25% and 50%, respectively.

### Gene expression analysis

Two data sources were used for gene expression analysis. For processing the data from the FlyAtlas project [55], we used genomic coordinates from Affymetrix Drosophila Genome Tiling 2.0R Array release 5.33 (gene names and sequences from FlyBase 5.3 were used to design this array release). We also used FlyBase High Throughput Expression Pattern Data (Gelbart, W.M., Emmert, D.B., 2013, in FlyBase, http://flybase.org/static_pages/feature/previous/articles/2013_05/rna-seq_bulk.html, description: http://flybase.org/reports/FBrf0221009.html). Genomic coordinates of both expression datasets were converted so as to match the FlyBase release 5.50.

### Data processing and HMM clustering

Data from the fly modENCODE project were extensively used in our analyses and appropriate datasets are listed in the Table S3 (modENCODEFiles.csv). Custom scripts in R language [73] were used for data processing. To perform principal component analysis (PCA) on chromatin protein profiles, we used *prcomp* function in R. Since we analyzed pre-normalized log2-converted ChIP-chip data, no scaling was applied. Data clustering was done using *RHmm* library package in R [74]. To simplify comparisons and statistical analysis of log2-scaled ChIP-chip data, we subdivided the genome into consecutive 200 bp-long fragments using a non-overlapping sliding window method. Protein localization data were processed using HMM-based script with multivariate normal emission distributions, determined from the Baum–Welch algorithm, applying two first principal components to a set of 12 reference interband regions. Four states were chosen as appropriate, based on the Calinski-Harabasz criterion [75], [76] applied for 14 models trained on X-chromosomal sequences with the number of states ranging from 2 to 15 (Fig. S14). Regions of enrichment for 12 interband regions were determined from the Viterbi path. Interbands were identified as one of these four HMM states (cyan), as all 12 P-element insertions previously mapped in interbands [32] were within the cyan domains. All scripts used to download and process modEncode datasets and to obtain the 4-state chromatin model can be found in the File S3 (hmm.tar.gz). The final file for loading our 4-state model data as a genome browser track is provided in the File S1 (hmm4.bed.gz).

## Supporting Information

Figure S1 **Cytological identification of the interbands in the 100B region of the** ***Drosophila melanogaster*** **3R chromosome. A, D** – Comparison between Bridges revised map [39] (**A**) and Electron Microscopic map of the region 100A (**D**) (scale represents three micra). Increased part of the interbands proximally and distally to the 100B3 small grey band is shown in the rectangle. **B, C** - Immunofluorescent localization of CHRIZ in the region. The interbands 100B1-2/100B3 and 100B3/100B4-5 are marked by asterisks. **E, F** - FISH localization of the DNA containing a fragment of the *dco* gene (arrow in **F**), phase contrast as a control (**C**).
(TIF)

Figure S2 **Heatmap showing correlation between protein distributions along the X chromosome.** Spearman correlation matrix between protein binding data on the X chromosome (ChIP-chip, modENCODE Consortium, 2010). Pairwise correlation values are presented and color-coded according to the color map shown on the bottom left. Spearman correlation distances are illustrated by the dendrogram on the left of the graph. The cluster of proteins to be analyzed in more detail was identified using the appropriate stopping rule [40] and is highlighted as a green frame.
(TIFF)

Figure S3 **Localization of proteins and genomic features (fly modENCODE) around the interbands 100B1-2 - 100B4-5.** Dashed red vertical lines show the edges of cyan state chromatin in the region which conditionally reflect the location of the interbands. **A –** gene map (RefSeq Genes). **B** – Localization of promoter broad, unknown and peacked types according to Hoskins et al. (2011) [42] (light green, red and blue rectangles) and probes for FISH (black). **C** – Localization of 4-state chromatin types according to the algorithm developed in this paper. Only cyan and green chromatin types map to this genome region. **D –** Five-state chromatin types in Kc cells by Filion et al. [20]. **E** - 9-chromatin states in S2 cells by Kharchenko et al. [22]. Chromatin state 1 is marked with red. **F** - 9-chromatin states in BG3 cells by Kharchenko et al. [22]. Chromatin state 1 is marked with red. **G –** Nucleosome density according to Henikoff et al. [43]. Peaks above the axis reflect high density and those below axis denote low nucleosome density. **H** – Localization of histone H1 dips in Kc cells by Braunschweig et al. [44]. Black horizontal bars indicate the genomic regions with low Histone H1 binding. **I** - DNAse I hypersensitivity sites (high magnitude DHS - vertical lines) in S2, BG3, and Kc cells by Kharchenko et al. [22]. **J** - ORC2-binding sites in S2, BG3, Kc cells and salivary glands by Eaton et al. [45], Sher et al. [46]. **K –** Enrichment profiles of NSL complex components: NSL1 binding profile from salivary glands by Raja et al. [47], NSL3 in S2 cells by Lam et al. [48], NSL1 in S2 cells by Feller et al. [49]. **L –** Enrichment regions of various proteins specific for interbands and active chromatin (fly modENCODE). The list of interband-specific proteins is taken from Demakov et al. [28] and Vatolina et al. [27].
(TIF)

Figure S4 **Localization of proteins and genomic features (modENCODE) around the interband 1A8/1B1-2.** Red dotted vertical lines are according to edges of cyan state chromatin in the region which conditionally reflect the location of the interbands. **A –** gene map (RefSeq Genes). **B** – Localization of pICon3C(1A) reference transposon, of promoter broad and unknown types according to Hoskins et al. (2011) [42] (light green and red rectangles). **C** – Localization of 4-state chromatin types according to the algorithm developed in this paper. Only cyan and green chromatin types map to this genome region. **D –** Five-state chromatin types in Kc cells by Filion et al. [20]. **E** - 9-chromatin states in S2 cells by Kharchenko et al. [22]. Chromatin state 1 is marked with red. **F** - 9-chromatin states in BG3 cells by Kharchenko et al. [22]. Chromatin state 1 is marked with red. **G –** Nucleosome density according to Henikoff et al. [43]. Peaks above the axis reflect high density and those below axis denote low nucleosome density. **H** – Localization of histone H1 dips in Kc cells by Braunschweig et al. [44]. Black horizontal bars indicate the genomic regions with low Histone H1 binding. **I** - DNAse I hypersensitivity sites (high magnitude DHS - vertical lines) in S2, BG3, and Kc cells by Kharchenko et al. [22]. **J** - ORC2-binding sites in S2, BG3, Kc cells and salivary glands by Eaton et al. [45], Sher et al. [46]. **K –** Enrichment profiles of NSL complex components: NSL1 binding profile from salivary glands by Raja et al. [47], NSL3 in S2 cells by Lam et al. [48], NSL1 in S2 cells by Feller et al. [49]. **L –** Enrichment regions of various proteins specific for interbands and active chromatin (fly modENCODE). The list of interband-specific proteins is taken from Demakov et al. [28] and Vatolina et al. [27].
(TIF)

Figure S5 **Localization of proteins and genomic features (modENCODE) around the interband 10A7/10A8-9.** Red dashed vertical lines are according to edges of cyan state chromatin in the region which conditionally reflect the location of the interbands. **A –** gene map (RefSeq Genes) and position of the reference transposon insertion P{EPgy2}EY09320 (arrow). **B** – Localization of promoter broad, peacked and unknown types according to Hoskins et al. (2011) [42] (light green blue and red rectangles). **C** – Localization of 4-state chromatin types according to the algorithm developed in this paper. Only cyan and green chromatin types map to this genome region. **D –** Five-state chromatin types in Kc cells by Filion et al. [20]. **E** - 9-chromatin states in S2 cells by Kharchenko et al. [22]. Chromatin state 1 is marked with red. **F** - 9-chromatin states in BG3 cells by Kharchenko et al. [22]. Chromatin state 1 is marked with red. **G –** Nucleosome density according to Henikoff et al. [43]. Peaks above the axis reflect high density and those below axis denote low nucleosome density. **H** – Localization of histone H1 dips in Kc cells by Braunschweig et al. [44]. Black horizontal bars indicate the genomic regions with low Histone H1 binding. **I** - DNAse I hypersensitivity sites (high magnitude DHS - vertical lines) in S2, BG3, and Kc cells by Kharchenko et al. [22]. **J** - ORC2-binding sites in S2, BG3, Kc cells and salivary glands by Eaton et al. [45], Sher et al. [46]. **K –** Enrichment profiles of NSL complex components: NSL1 binding profile from salivary glands by Raja et al. [47], NSL3 in S2 cells by Lam et al. [48], NSL1 in S2 cells by Feller et al. [49]. **L –** Enrichment regions of various proteins specific for interbands and active chromatin (fly modENCODE). The list of interband-specific proteins is taken from Demakov et al. [28] and Vatolina et al. [27].
(TIF)

Figure S6 **FISH localization of transposon insertions in the polytene chromosome interband regions 10A1-2/10A3 (A, D and G), 10A3/10A4-5 (D, E and H) and 10A7/10A8-9 (C, F and I).** **A - C –** overlay of FISH signal (green) and phase contrast. **D – F –** overlay of FISH signal (green), phase contrast and DAPI (blue). **G – I –** overlay of FISH signal (green) and DAPI (blue). Arrows point to the FISH signals in polytene chromosome regions.
(TIF)

Figure S7 **Box-and-whiskers diagram reflecting activity of genes located in the set of select 32 interbands (A) and intercalary heterochromatin bands (B).** To plot this diagram, data from Chintapalli et al. [55] were used. The list of tissues and organs is shown along the *X* axis. Mean mRNA expression (log_10_ scale) is shown on the *Y* axis. Median value is shown as thick black line; open boxes represent the data between 25 and 75 percentile (50% of data points). Whiskers extend to the most extreme data points which are no more than 1.5 times the length of the box away from the box. Separate circles represent outliers.
(TIFF)

Figure S8 **Box-and whiskers diagram reflecting activity of genes located in the cyan (A) and magenta (B) chromatin types in the whole genome.** Labeling is the same as in the Figure S7.
(TIFF)

Figure S9 **Box-and-whiskers diagram showing mean expression of genes located in the 32 interband chromatin according to modENCODE RNA-seq data.** The list of datasets on temporal, tissue, treatment and cell line expression is given below the *x* axis. Y axis shows gene expression levels (RPKM in log_10_ scale) (according to [56] and S. Celniker group). Median value is shown as a thick black line; open boxes represent the data between 25 and 75 percentile (50% of data points). Whiskers extend to the most extreme data points which are no more than 1.5 times the length of the box away from the box. Separate circles represent outliers.
(TIFF)

Figure S10 **Box-and-whiskers diagram reflecting mean activity of genes located in IH bands according to modENCODE RNA-seq data.** Labeling is the same as in the **Figure S9**.
(TIFF)

Figure S11 **Box-and-whiskers diagram reflecting mean activity of genes located in the cyan type of chromatin in the whole genome according to modENCODE RNA-seq data.** Explanations as in the **Fig. S9**.
(TIFF)

Figure S12 **Box-and-whiskers diagram reflecting mean activity of genes located in magenta type of chromatin in whole genome according to modENCODE RNA-Seq data.** Explanations as in the **Fig. S9**.
(TIFF)

Figure S13 **Different types of genome organization domains described for the region 9F13 – 10B3.** **A -** Genomic coordinates around the 10A1-2 and 10B1-2 bands (red dashed lines). **B -** Track showing our four-state chromatin types. The span of 10A1-2 the 10A1-2 and 10B1-2 bands is indicated above the track. **C** - Map of five-state chromatin types in Kc cells by Filion et al. [20]. **D** - Position of 9 chromatin states in S2 cells by Kharchenko et al. [22]. **E** - Position of 9 chromatin states in BG3 cells by Kharchenko et a. [22]. **F** - Physical domains by Sexton et al. [67]. “Active” domain is shown in red. It indicates the transcriptionally inert IH band 10A1-2 and overlaps with theexpressed region (a series of grey bands and interbands) encompassing housekeeping genes. “Null” domain (shown in black) co-localizes with the transcriptionally silent band 10B1-2. **G** - Physical domains by Hou et al. [68]. **H** - Select genes from FlyBase which according to Weber, Hurst [69] are housekeeping. **I** - Select genes from FlyBase referenced as housekeeping in Feller et al. [49]. **J** - FlyBase genes. Genes that we classify as housekeeping are shown in red. **K** - NSL1 enrichment profile in S2 cells according to Feller et al. [49]. **L** - NSL1 enrichment in salivary glands according to Raja et al. [47]. **M** - NSL3 enrichment in S2 cells according to Lam et al. [48].
(TIF)

Figure S14 **Values of Calinski-Harabasz criterion at different numbers of states used for HMM clustering of** ***D. melanogaster*** **X chromosome sequences.**
(TIF)

Text S1 **Identification of a new set of interbands in** ***Drosophila melanogaster*** **polytene chromosomes.**
(DOC)

Table S1 **Lists of genes, whose 5′-ends map to the cytologically defined interbands and genes located completely in the intercalary heterochromatin bands.**
(XLS)

Table S2 **Coordinates and descriptions of probes selected for FISH mapping on polytene chromosomes (release 5.12).**
(DOC)

Table S3 **The list of ChIP-chip protein binding data sets used for clusterization (modENCODE Consortium, 2010).**
(XLS)

File S1 **Track of 4-state chromatin model developed in this study.**
(GZ) Click here for additional data file.

File S2 **Track of head-to-head oriented genes.**
(GZ) Click here for additional data file.

File S3 **Scripts in R language for building 4-state chromatin model.**
(GZ) Click here for additional data file.
