# Supplementary figures and images for: Genetic Organization of Interphase Chromosome Bands and Interbands in Drosophila melanogaster
Source: PLoS One. 2014 Jul 29;9(7):e101631. doi: 10.1371/journal.pone.0101631 (PMC4114487; doi:10.1371/journal.pone.0101631)

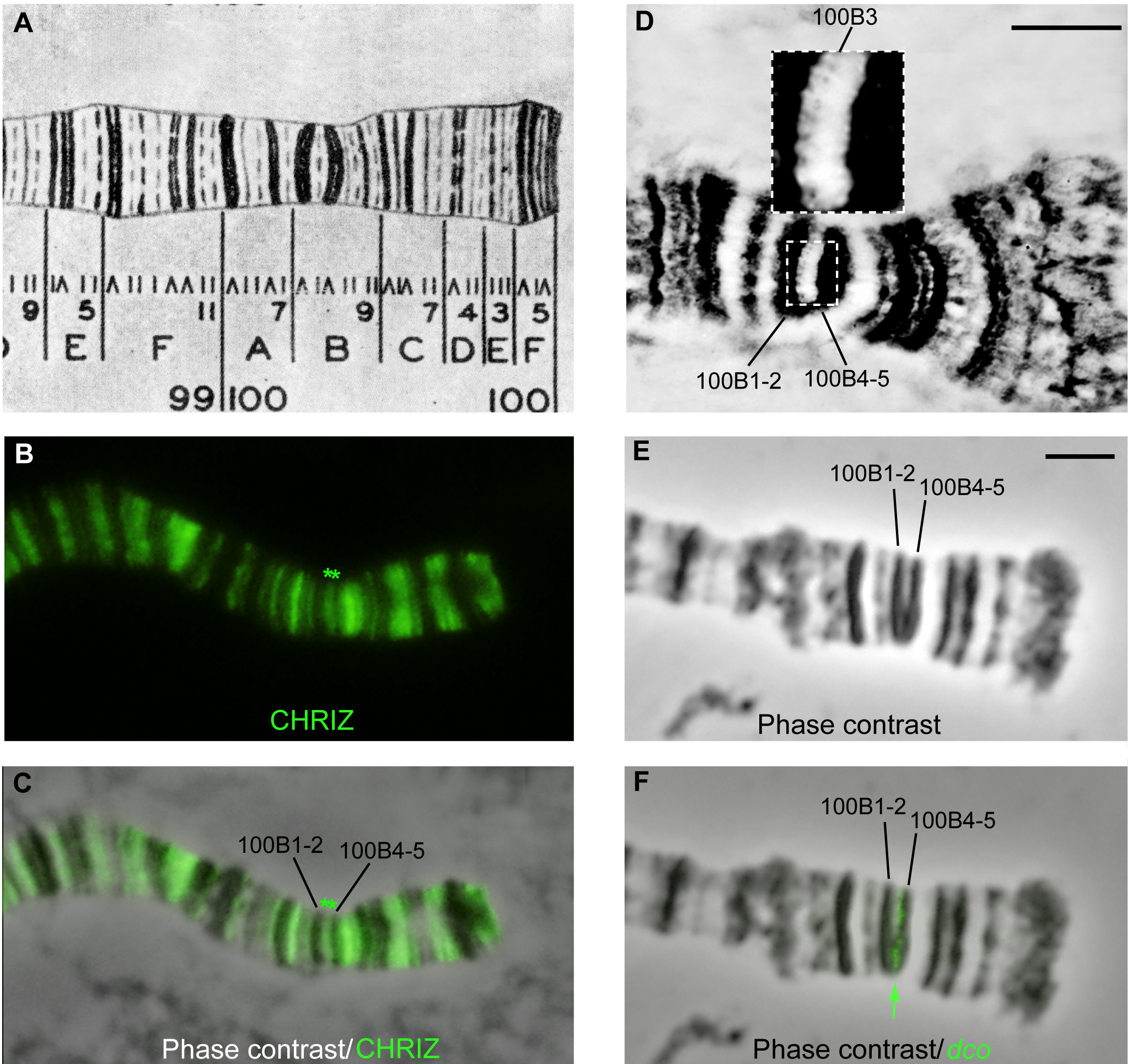

Supplement: Figure S1 — Cytological identification of the interbands in the 100B region of the Drosophila melanogaster 3R chromosome. A, D – Comparison between Bridges revised map [39] (A) and Electron Microscopic map of the region 100A (D) (scale represents three micra). Increased part of the interbands proximally and distally to the 100B3 small grey band is shown in the rectangle. B, C - Immunofluorescent localization of CHRIZ in the region. The interbands 100B1-2/100B3 and 100B3/100B4-5 are marked by asterisks. E, F - FISH localization of the DNA containing a fragment of the dco gene (arrow in F), phase contrast as a control (C). (TIF) [file pone.0101631.s001.tif]

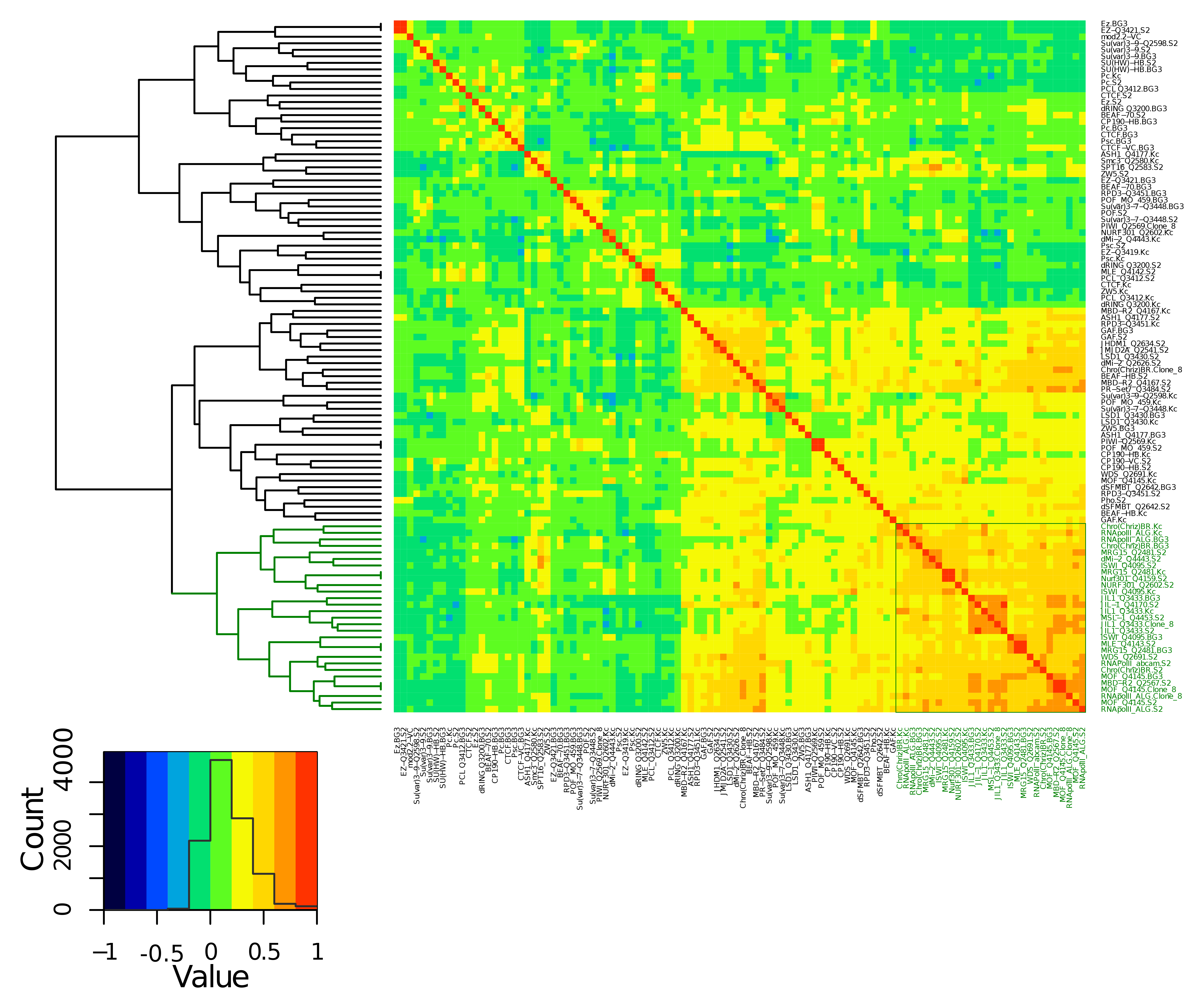

Supplement: Figure S2 — Heatmap showing correlation between protein distributions along the X chromosome. Spearman correlation matrix between protein binding data on the X chromosome (ChIP-chip, modENCODE Consortium, 2010). Pairwise correlation values are presented and color-coded according to the color map shown on the bottom left. Spearman correlation distances are illustrated by the dendrogram on the left of the graph. The cluster of proteins to be analyzed in more detail was identified using the appropriate stopping rule [40] and is highlighted as a green frame. (TIFF) [file pone.0101631.s002.tiff]

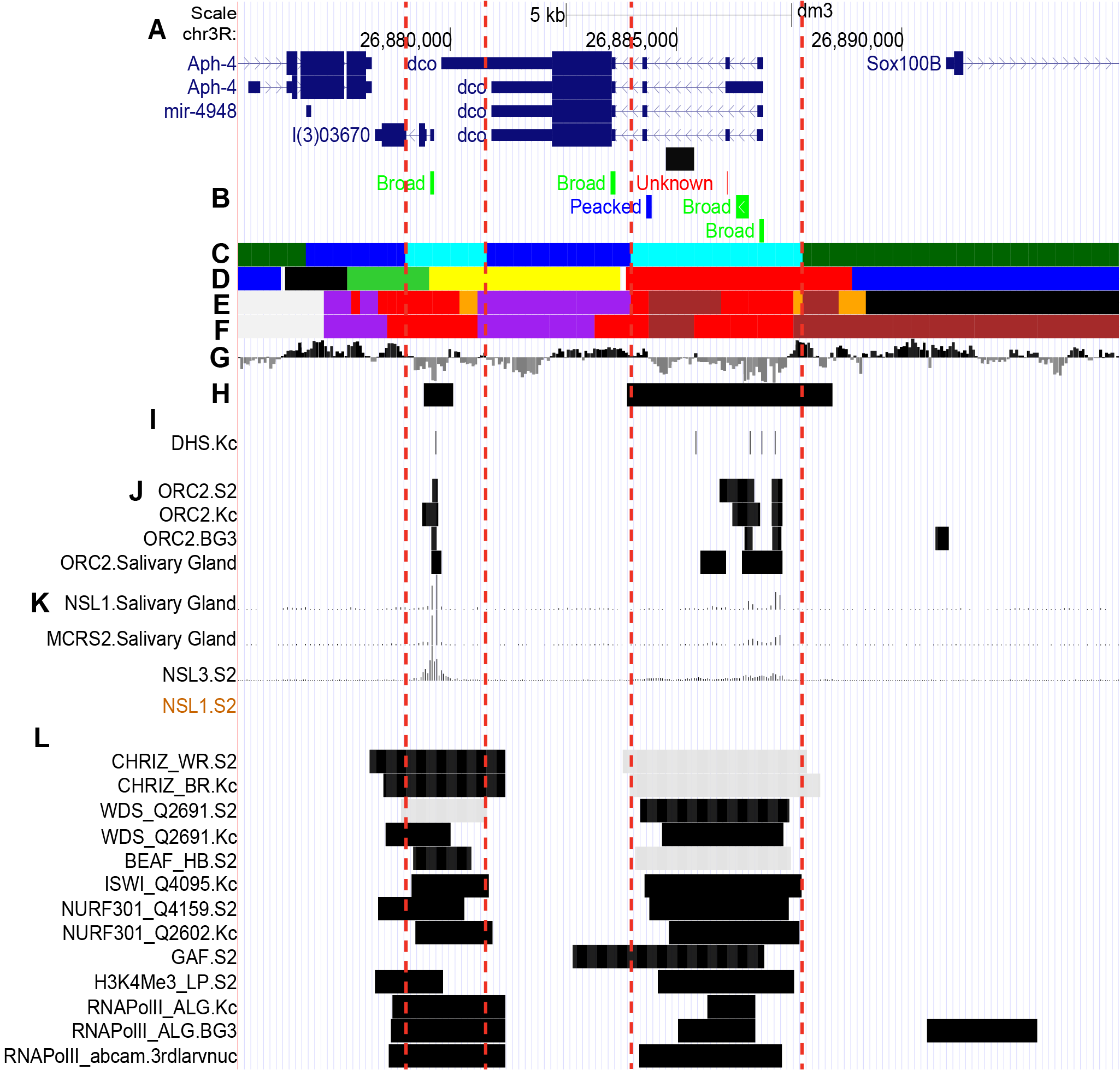

Supplement: Figure S3 — Localization of proteins and genomic features (fly modENCODE) around the interbands 100B1-2 - 100B4-5. Dashed red vertical lines show the edges of cyan state chromatin in the region which conditionally reflect the location of the interbands. A – gene map (RefSeq Genes). B – Localization of promoter broad, unknown and peacked types according to Hoskins et al. (2011) [42] (light green, red and blue rectangles) and probes for FISH (black). C – Localization of 4-state chromatin types according to the algorithm developed in this paper. Only cyan and green chromatin types map to this genome region. D – Five-state chromatin types in Kc cells by Filion et al. [20]. E - 9-chromatin states in S2 cells by Kharchenko et al. [22]. Chromatin state 1 is marked with red. F - 9-chromatin states in BG3 cells by Kharchenko et al. [22]. Chromatin state 1 is marked with red. G – Nucleosome density according to Henikoff et al. [43]. Peaks above the axis reflect high density and those below axis denote low nucleosome density. H – Localization of histone H1 dips in Kc cells by Braunschweig et al. [44]. Black horizontal bars indicate the genomic regions with low Histone H1 binding. I - DNAse I hypersensitivity sites (high magnitude DHS - vertical lines) in S2, BG3, and Kc cells by Kharchenko et al. [22]. J - ORC2-binding sites in S2, BG3, Kc cells and salivary glands by Eaton et al. [45], Sher et al. [46]. K – Enrichment profiles of NSL complex components: NSL1 binding profile from salivary glands by Raja et al. [47], NSL3 in S2 cells by Lam et al. [48], NSL1 in S2 cells by Feller et al. [49]. L – Enrichment regions of various proteins specific for interbands and active chromatin (fly modENCODE). The list of interband-specific proteins is taken from Demakov et al. [28] and Vatolina et al. [27]. (TIF) [file pone.0101631.s003.tif]

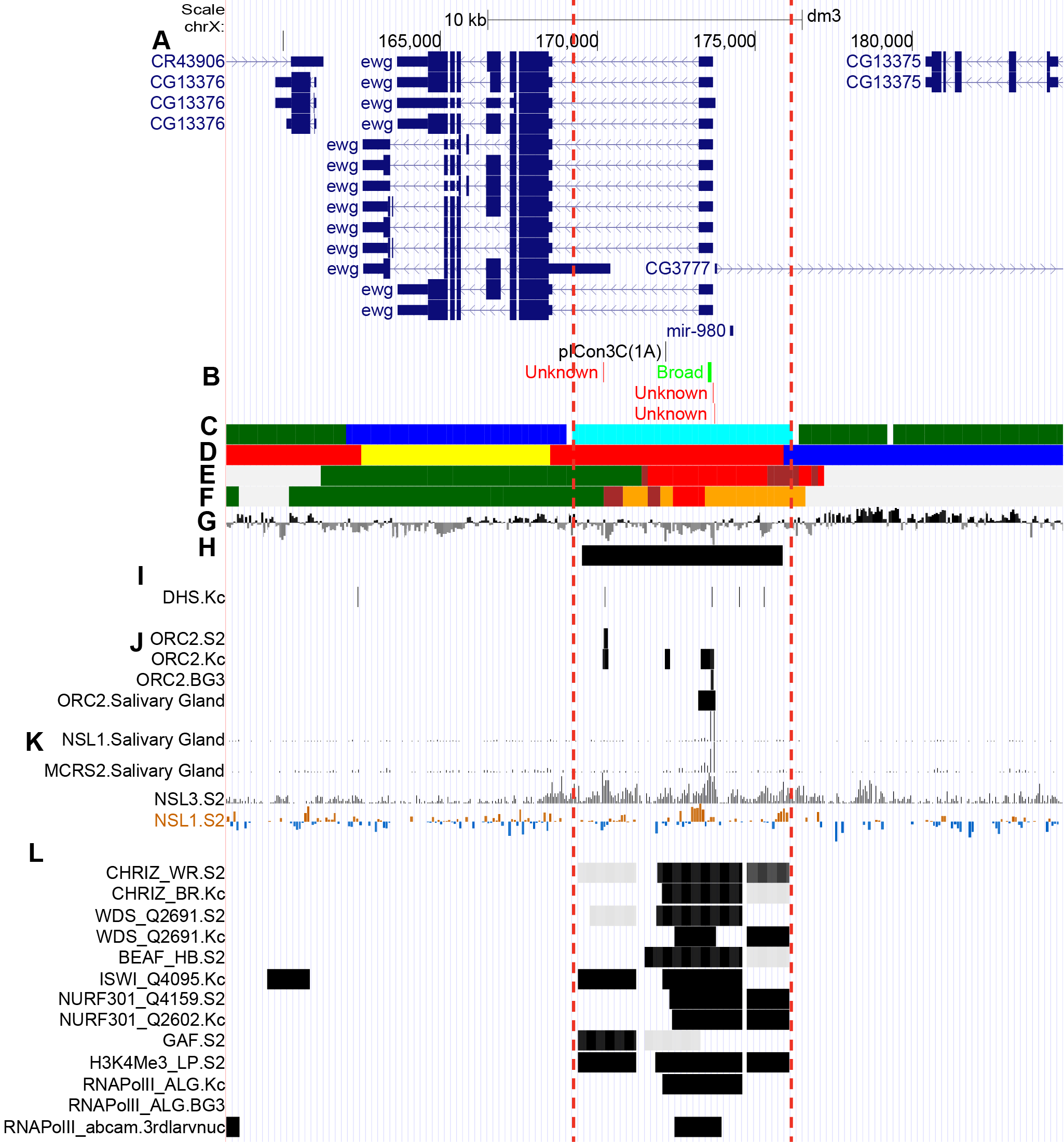

Supplement: Figure S4 — Localization of proteins and genomic features (modENCODE) around the interband 1A8/1B1-2. Red dotted vertical lines are according to edges of cyan state chromatin in the region which conditionally reflect the location of the interbands. A – gene map (RefSeq Genes). B – Localization of pICon3C(1A) reference transposon, of promoter broad and unknown types according to Hoskins et al. (2011) [42] (light green and red rectangles). C – Localization of 4-state chromatin types according to the algorithm developed in this paper. Only cyan and green chromatin types map to this genome region. D – Five-state chromatin types in Kc cells by Filion et al. [20]. E - 9-chromatin states in S2 cells by Kharchenko et al. [22]. Chromatin state 1 is marked with red. F - 9-chromatin states in BG3 cells by Kharchenko et al. [22]. Chromatin state 1 is marked with red. G – Nucleosome density according to Henikoff et al. [43]. Peaks above the axis reflect high density and those below axis denote low nucleosome density. H – Localization of histone H1 dips in Kc cells by Braunschweig et al. [44]. Black horizontal bars indicate the genomic regions with low Histone H1 binding. I - DNAse I hypersensitivity sites (high magnitude DHS - vertical lines) in S2, BG3, and Kc cells by Kharchenko et al. [22]. J - ORC2-binding sites in S2, BG3, Kc cells and salivary glands by Eaton et al. [45], Sher et al. [46]. K – Enrichment profiles of NSL complex components: NSL1 binding profile from salivary glands by Raja et al. [47], NSL3 in S2 cells by Lam et al. [48], NSL1 in S2 cells by Feller et al. [49]. L – Enrichment regions of various proteins specific for interbands and active chromatin (fly modENCODE). The list of interband-specific proteins is taken from Demakov et al. [28] and Vatolina et al. [27]. (TIF) [file pone.0101631.s004.tif]

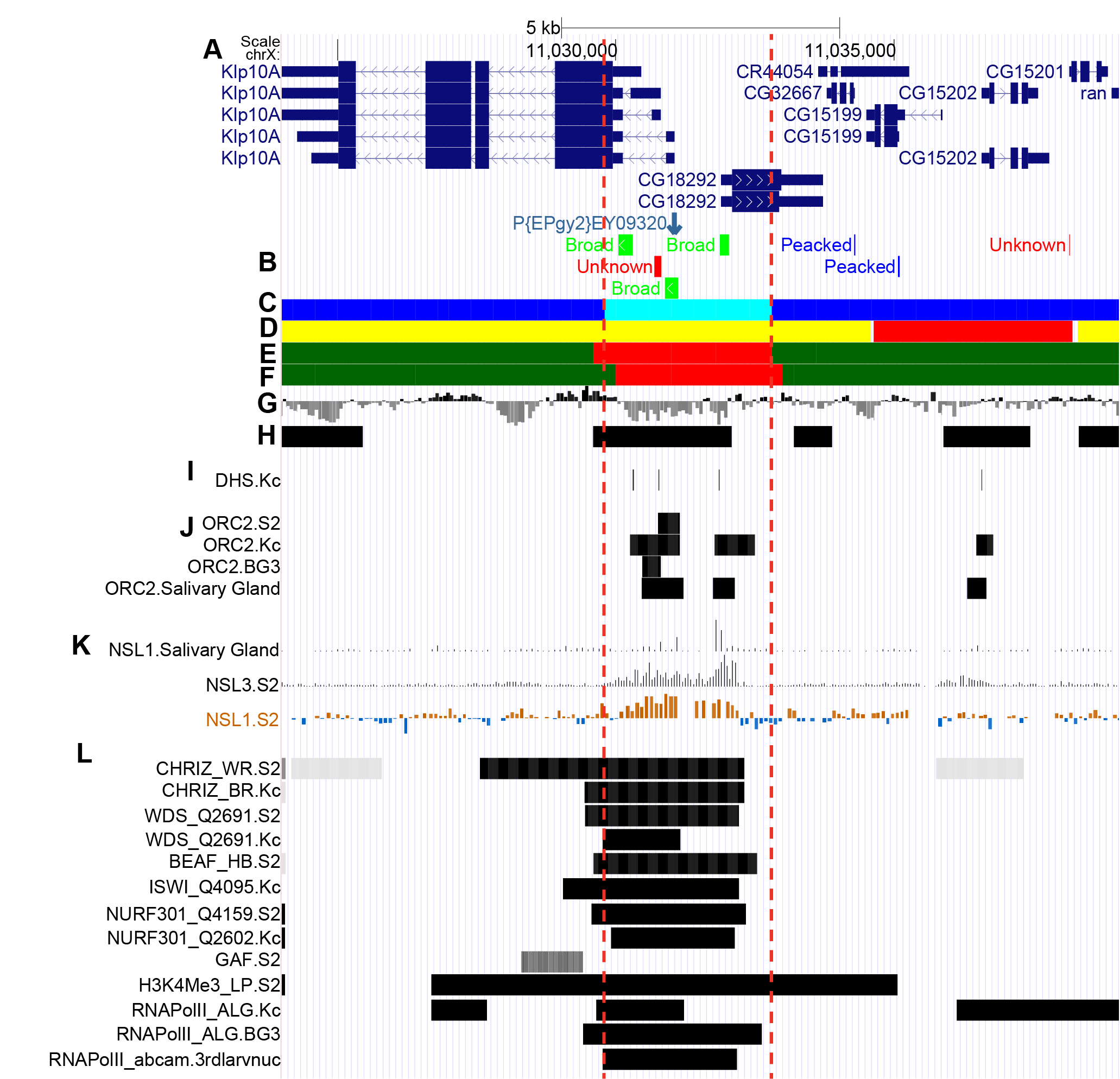

Supplement: Figure S5 — Localization of proteins and genomic features (modENCODE) around the interband 10A7/10A8-9. Red dashed vertical lines are according to edges of cyan state chromatin in the region which conditionally reflect the location of the interbands. A – gene map (RefSeq Genes) and position of the reference transposon insertion P{EPgy2}EY09320 (arrow). B – Localization of promoter broad, peacked and unknown types according to Hoskins et al. (2011) [42] (light green blue and red rectangles). C – Localization of 4-state chromatin types according to the algorithm developed in this paper. Only cyan and green chromatin types map to this genome region. D – Five-state chromatin types in Kc cells by Filion et al. [20]. E - 9-chromatin states in S2 cells by Kharchenko et al. [22]. Chromatin state 1 is marked with red. F - 9-chromatin states in BG3 cells by Kharchenko et al. [22]. Chromatin state 1 is marked with red. G – Nucleosome density according to Henikoff et al. [43]. Peaks above the axis reflect high density and those below axis denote low nucleosome density. H – Localization of histone H1 dips in Kc cells by Braunschweig et al. [44]. Black horizontal bars indicate the genomic regions with low Histone H1 binding. I - DNAse I hypersensitivity sites (high magnitude DHS - vertical lines) in S2, BG3, and Kc cells by Kharchenko et al. [22]. J - ORC2-binding sites in S2, BG3, Kc cells and salivary glands by Eaton et al. [45], Sher et al. [46]. K – Enrichment profiles of NSL complex components: NSL1 binding profile from salivary glands by Raja et al. [47], NSL3 in S2 cells by Lam et al. [48], NSL1 in S2 cells by Feller et al. [49]. L – Enrichment regions of various proteins specific for interbands and active chromatin (fly modENCODE). The list of interband-specific proteins is taken from Demakov et al. [28] and Vatolina et al. [27]. (TIF) [file pone.0101631.s005.tif]

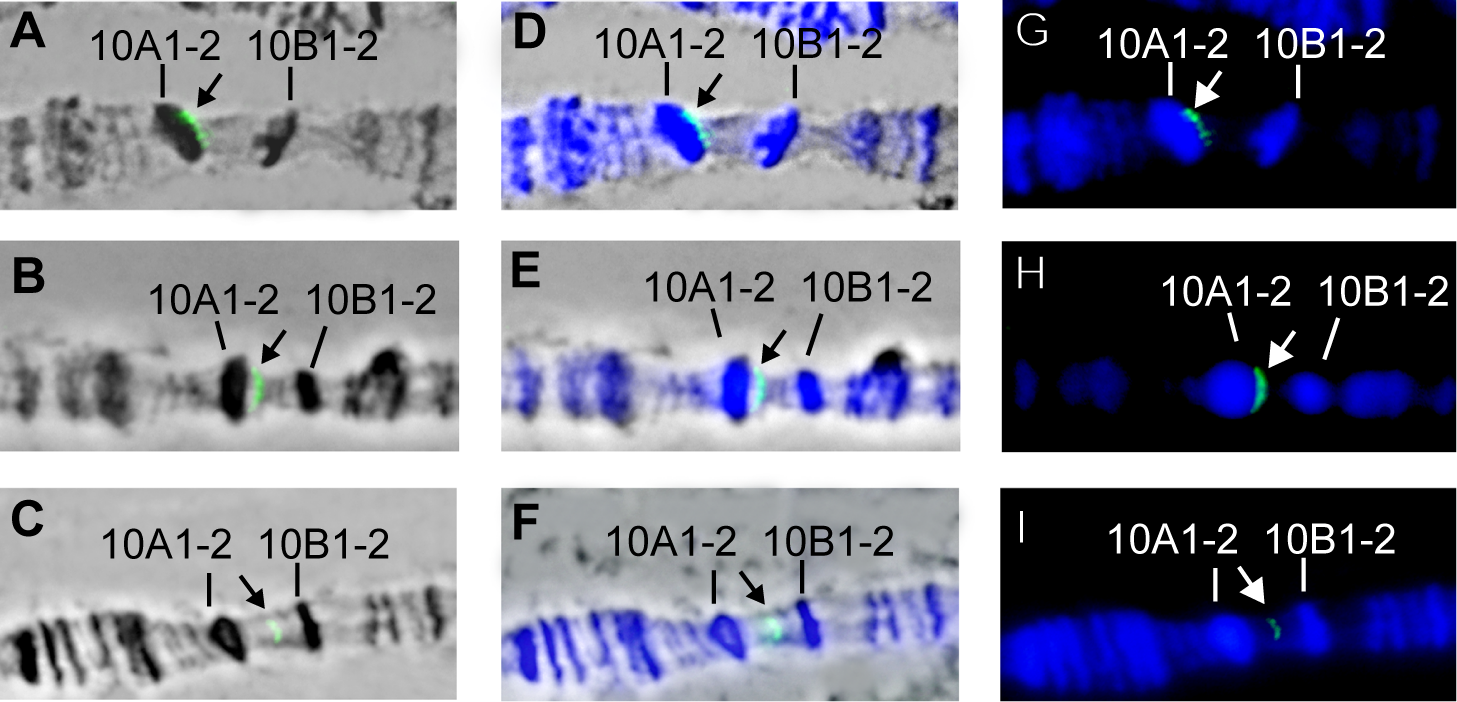

Supplement: Figure S6 — FISH localization of transposon insertions in the polytene chromosome interband regions 10A1-2/10A3 (A, D and G), 10A3/10A4-5 (D, E and H) and 10A7/10A8-9 (C, F and I). A - C – overlay of FISH signal (green) and phase contrast. D – F – overlay of FISH signal (green), phase contrast and DAPI (blue). G – I – overlay of FISH signal (green) and DAPI (blue). Arrows point to the FISH signals in polytene chromosome regions. (TIF) [file pone.0101631.s006.tif]

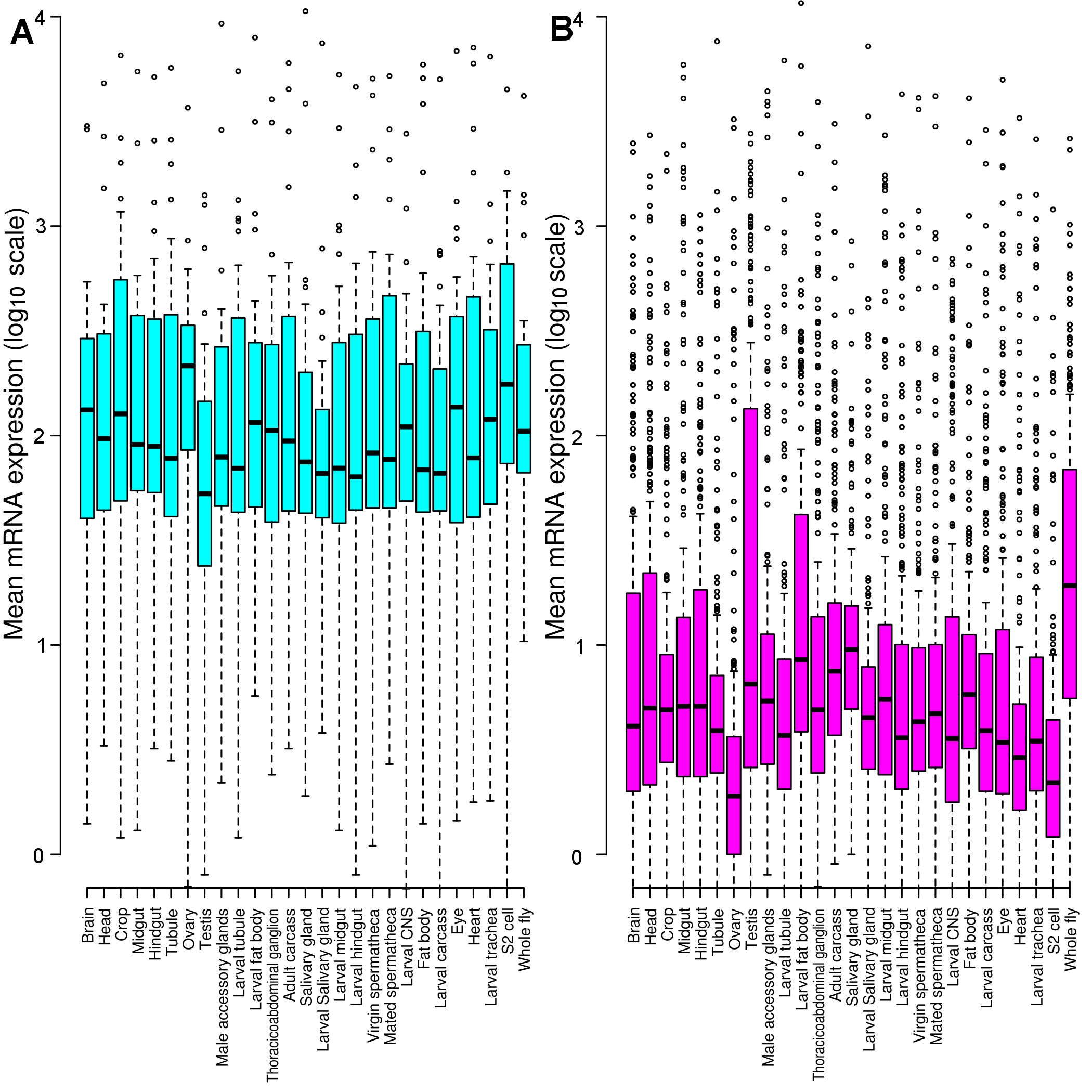

Supplement: Figure S7 — Box-and-whiskers diagram reflecting activity of genes located in the set of select 32 interbands (A) and intercalary heterochromatin bands (B). To plot this diagram, data from Chintapalli et al. [55] were used. The list of tissues and organs is shown along the X axis. Mean mRNA expression (log10 scale) is shown on the Y axis. Median value is shown as thick black line; open boxes represent the data between 25 and 75 percentile (50% of data points). Whiskers extend to the most extreme data points which are no more than 1.5 times the length of the box away from the box. Separate circles represent outliers. (TIFF) [file pone.0101631.s007.tiff]

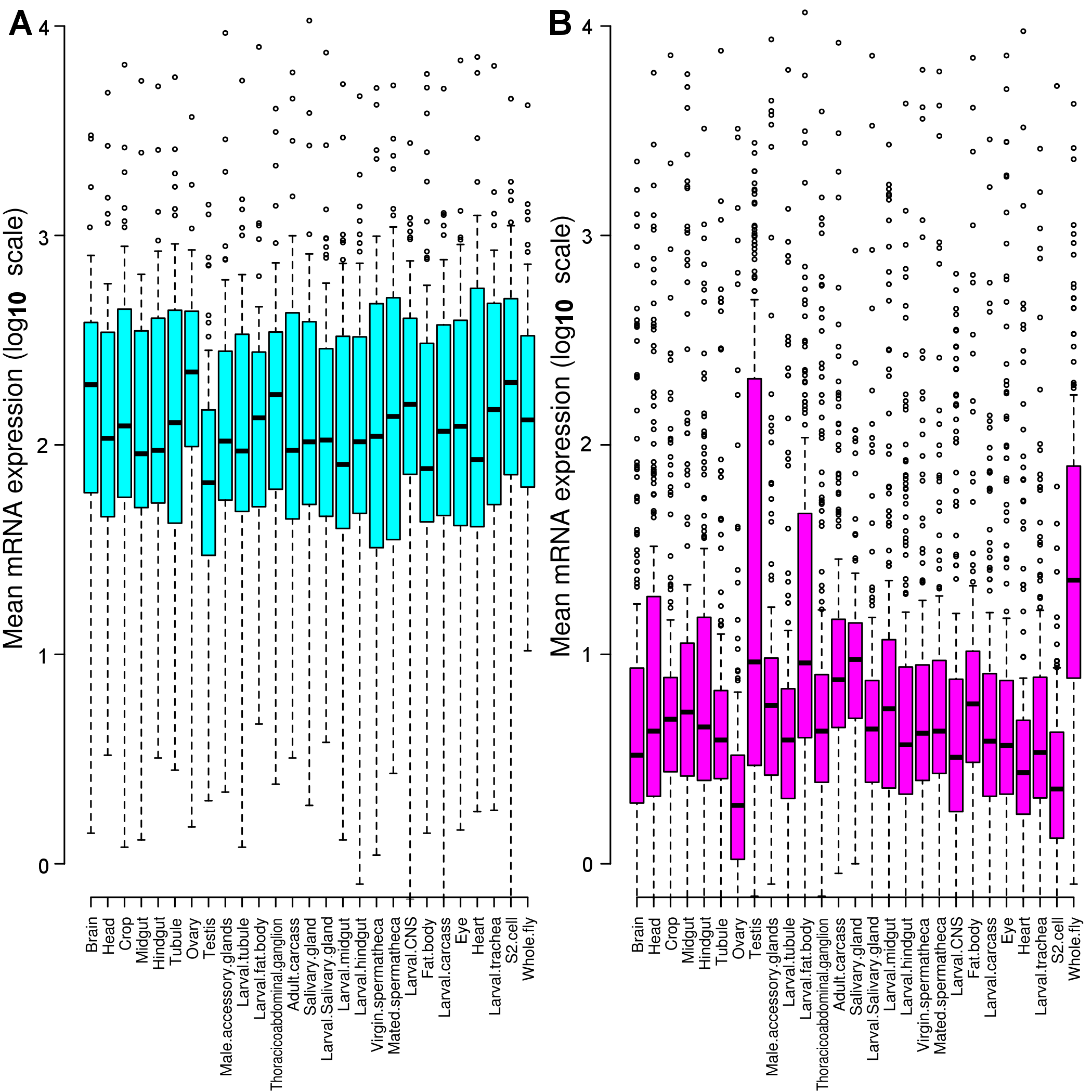

Supplement: Figure S8 — Box-and whiskers diagram reflecting activity of genes located in the cyan (A) and magenta (B) chromatin types in the whole genome. Labeling is the same as in the Figure S7. (TIFF) [file pone.0101631.s008.tiff]

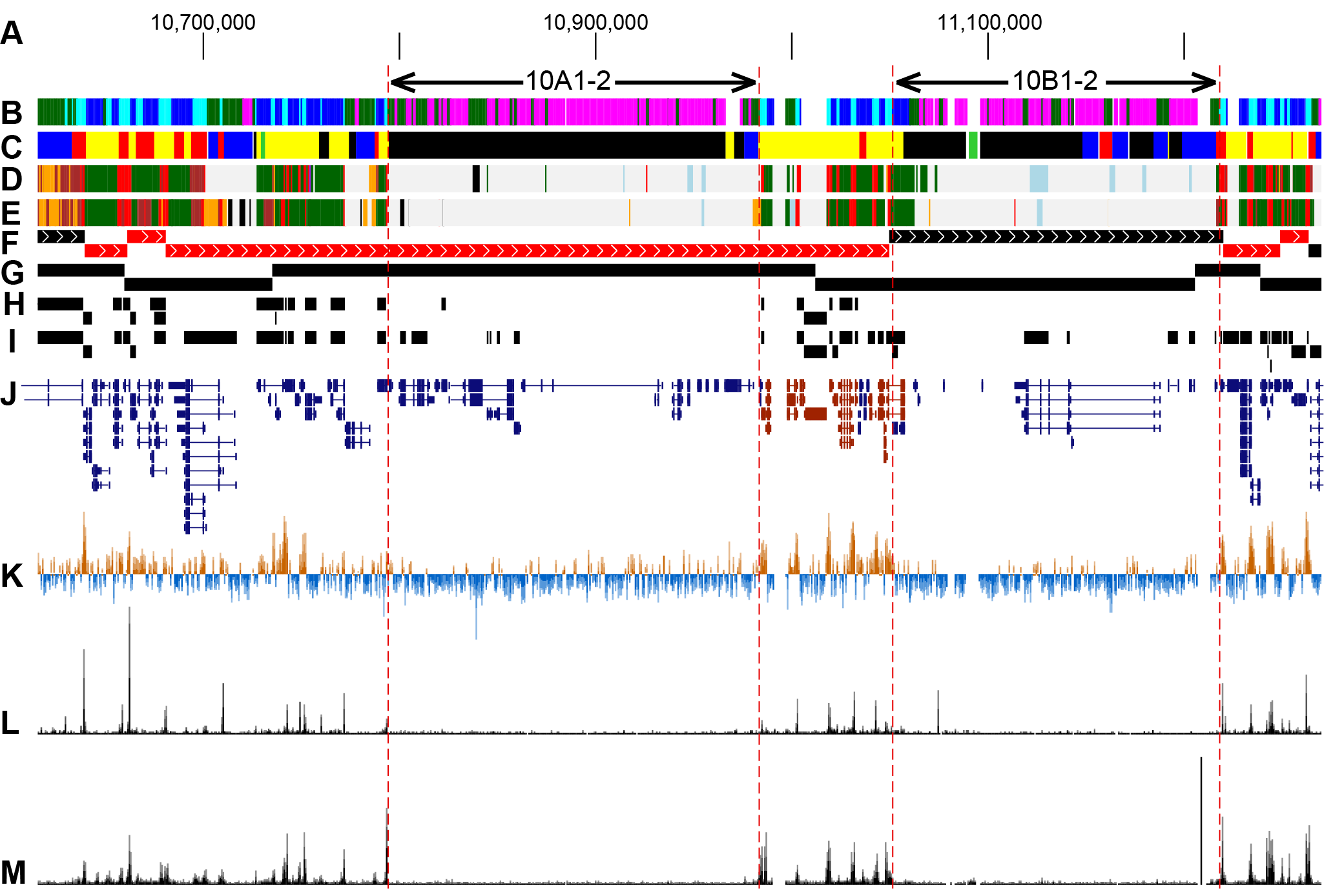

Supplement: Figure S13 — Different types of genome organization domains described for the region 9F13 – 10B3. A - Genomic coordinates around the 10A1-2 and 10B1-2 bands (red dashed lines). B - Track showing our four-state chromatin types. The span of 10A1-2 the 10A1-2 and 10B1-2 bands is indicated above the track. C - Map of five-state chromatin types in Kc cells by Filion et al. [20]. D - Position of 9 chromatin states in S2 cells by Kharchenko et al. [22]. E - Position of 9 chromatin states in BG3 cells by Kharchenko et a. [22]. F - Physical domains by Sexton et al. [67]. “Active” domain is shown in red. It indicates the transcriptionally inert IH band 10A1-2 and overlaps with theexpressed region (a series of grey bands and interbands) encompassing housekeeping genes. “Null” domain (shown in black) co-localizes with the transcriptionally silent band 10B1-2. G - Physical domains by Hou et al. [68]. H - Select genes from FlyBase which according to Weber, Hurst [69] are housekeeping. I - Select genes from FlyBase referenced as housekeeping in Feller et al. [49]. J - FlyBase genes. Genes that we classify as housekeeping are shown in red. K - NSL1 enrichment profile in S2 cells according to Feller et al. [49]. L - NSL1 enrichment in salivary glands according to Raja et al. [47]. M - NSL3 enrichment in S2 cells according to Lam et al. [48]. (TIF) [file pone.0101631.s013.tif]

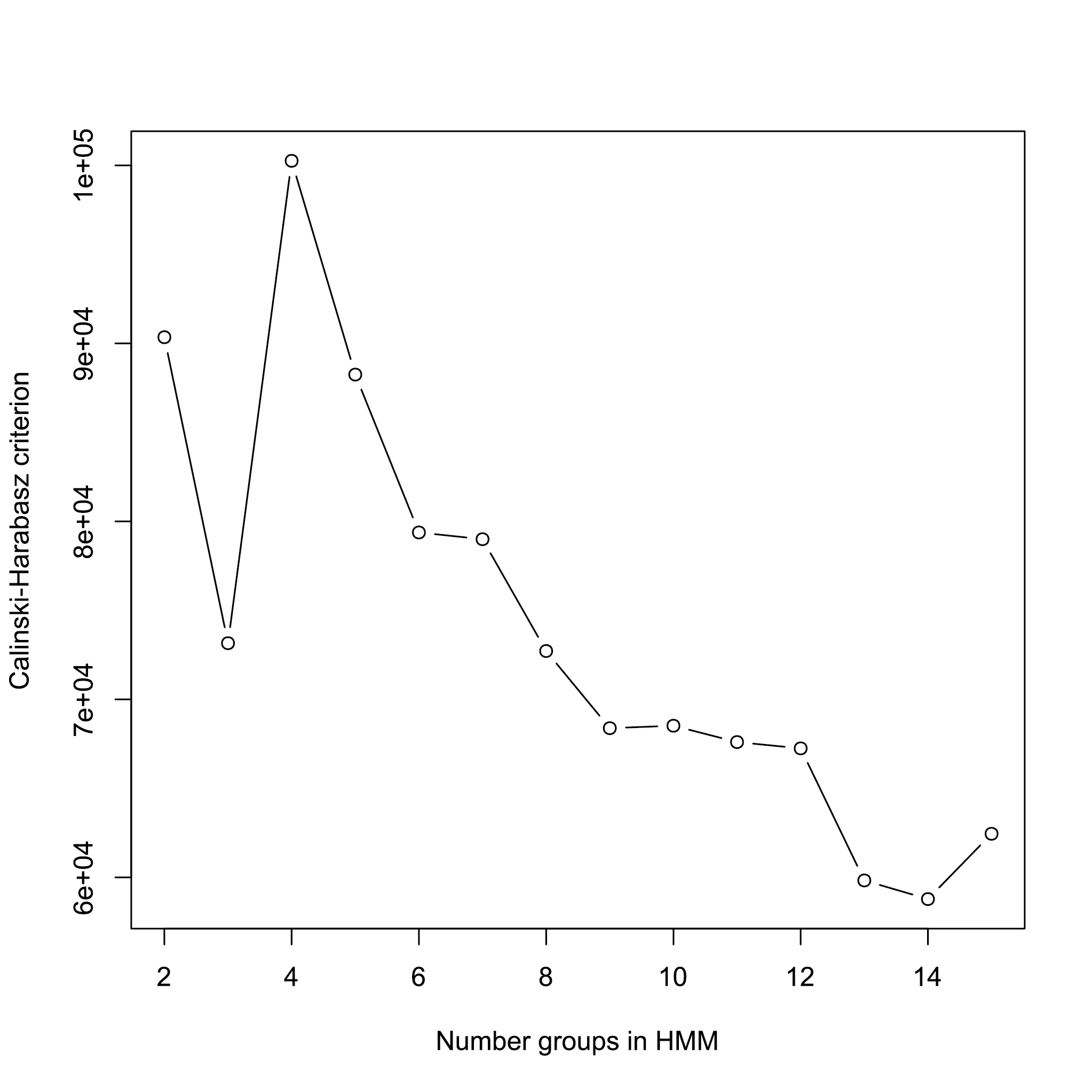

Supplement: Figure S14 — Values of Calinski-Harabasz criterion at different numbers of states used for HMM clustering of D. melanogaster X chromosome sequences. (TIF) [file pone.0101631.s014.tif]
