# Supplementary material for: Genetic Organization of Interphase Chromosome Bands and Interbands in Drosophila melanogaster
Source: PLoS One. 2014 Jul 29;9(7):e101631. doi: 10.1371/journal.pone.0101631 (PMC4114487; doi:10.1371/journal.pone.0101631)
Supplement: Text S1 — Identification of a new set of interbands in Drosophila melanogaster polytene chromosomes. (DOC) [file pone.0101631.s015.doc]

**Supplementary Text 1**

On Bridges map [35], the region 19Е1-4 is shown as two closely spaced doublets, 19Е1-2 and 19Е3-4. EM data suggest there are three dense bands separated by two interbands. 5’ end of *Cyp6v1* was established to map to the interband 19Е1-2/19E3-4 by FISH. This interband appropriately associates with an interband-specific mark, Chriz/CHROMATOR [26].

There are 4 bands and 3 interbands in the region 21D–E of the 2L polytene chromosome in agreement with Bridges’ [37] map: two dense bands 21D1-2 and 21 E1-2 on the flanks of the region and two thin loose bands between them. The banding pattern remains unaltered at different developmental stages or when different fixation methods are used [31]. All three interbands display pronounced Chriz/CHROMATOR-signal, with the strongest labeling found in the interband closest to 21D1-2. Two of these interbands, 21D1-2/21D3 and 21D4/21E1-2, encompass the fragments of *Nnf1b* and *dbe/PNUTS* genes, respectively.

In polytene chromosomes, the region 35D1-4 has two massive bands, – 35D1-2 and 35D3-4; these bands in fact correspond to the condensed and late-replicating material of IH [26]. Both on Bridges [37] and EM maps, the region between these two bands appears as a very thin genuine interband. FISH analysis puts the first intron of *lace* into this interband, which appropriately shows Chriz/CHROMATOR labeling [26].

On polytene maps, the region 56AB is depicted as having two doublets, 56А1-2 and 56В1-2 [38], as well as an interband 56А3 in between. Therefore, we believe, that bands 56А1-2 and 56В1-2 flank a typical interband, which encompasses the fragment of *prod/СG9304* gene. Consistently, this region appears Chriz/CHROMATOR-positive [26].

There are three doublets, - 58А1-2, 58А3-4 and 56В1-2, alternating with two interbands in the region 58AB of polytene maps [38]. EM analysis is consistent with this mapping. *CG9304* gene maps to the interband between the bands 58А3-4 and 56В1-2. Interband-specific protein Chriz/CHROMATOR is also found in this interband [26].

Two doublets, 70A1-2 and 70A4-5, are found in the region 70A1-5 on polytene chromosome map [36] with reticulated material in between, which harbors part of the *caps* gene and is bound by Chriz/CHROMATOR [26].
