# Supplementary material for: Genetic Organization of Interphase Chromosome Bands and Interbands in Drosophila melanogaster
Source: PLoS One. 2014 Jul 29;9(7):e101631. doi: 10.1371/journal.pone.0101631 (PMC4114487; doi:10.1371/journal.pone.0101631)
Supplement: Table S2 — Coordinates and descriptions of probes selected for FISH mapping on polytene chromosomes (release 5.12). (DOC) [file pone.0101631.s017.doc]

| Probes | Localization of probe on the cytological map (see Results) | Description of the probe | Primers | Coordinates of probe |
| --- | --- | --- | --- | --- |
| Nrg | Interband 7F1-2/ 7F3-4 | 5’ noncoding region and part of the first intron of the *Neuroglian (*[*CG1634*](http://flybase.org/cgi-bin/gbrowse/dmel/?dontadjust=1&name=FBgn0264975)*)* gene | 5’-GTGATGCCACCCCGCATTCC -3’ 5’- GCGTGGCTTTTGGCGTCCTC -3’ | Х: 8411355.. 8412090 |
| Cyp6v1 | Interband 19E1-2/ 19E3-4 | 5’ noncoding region of the *Cyp6v1*(*CG1829*) gene | 5’- GCGGAGGAGCGCCAAAACGATA -3’ 5’- GGTGGCTGAATCGTGGAGGGAT -3’ | X: 20528760.. 20529408 |
| Nnf1b | Interband 21D1-2/ 21D3 | Intergenic region, 5’ noncoding region, the first coding exon, intron and a part of the second coding exon of the *Nnf1b (*[*CG31658*](http://flybase.org/cgi-bin/gbrowse/dmel/?dontadjust=1&name=FBgn0051658)*)* gene | 5’- GCAAGTTCCTTCGCCGATGCG -3’ 5’- TCTCCACGTCGTTGCCGGAG -3’ | 2L: 810299.. 810960 |
| dbe/PNUTS | Interband 21D4/ 21E1-2 | Intergenic region and 5’ noncoding regions of the *PNUTS(*[*CG33526*](http://flybase.org/cgi-bin/gbrowse/dmel/?dontadjust=1&name=FBgn0053526)*)* and *dribble (*[*CG4258*](http://flybase.org/cgi-bin/gbrowse/dmel/?dontadjust=1&name=FBgn0020305)*)* genes | 5’- TGCACGCTTGTTTTGGCCAGG -3’ 5’- GGACATGCGGTTAGGTCCGC -3’ | 2L: 869820.. 870648 |
| lace | Interband 35D1-2/ 35D3-4 | Part of the first intron of the *lace (CG4162)* gene | 5’-CCTTTTCGCCGCGAGTGCTG-3’ 5’-CCGCTGCCTTCGGAGATTGC-3’ | 2L: 15499782.. 15500426 |
| prod | Interband 56A1-2/ 56B1-2 | Part of the first coding exon, the first intron and a part of the second exon of the *proliferation disrupter* ([*CG18608*](http://flybase.org/cgi-bin/gbrowse/dmel/?dontadjust=1&name=FBgn0014269)) gene | 5'- AGGCTGTGCCCGGATTGATGAC -3' 5'- AGGAGCTCTCCGACCGGCTAAA -3' | 2R: 14857720.. 14858368 |
| CG9304 | Interband 58A3-4/ 58B1-2 | Part of the second, third, fourth and part of the fifth coding exons; the second, third and fourth introns of the *CG*[*9304*](http://flybase.org/cgi-bin/gbrowse/dmel/?dontadjust=1&name=FBgn0014269) gene | 5'- TGCGCGTTCCACGTGTTCCTTT -3' 5'- CTCCCTGACAATGTGTGGCGCT -3' | 2R: 17862220.. 17862843 |
| caps | Interband 70A1-2/ 70A4-5 | Part of the first intron (in the 5’ noncoding region) of the *capricious (CG11282*) gene | 5'- GGCCAGAAAAACGCCTTGACAGC -3' 5'- GAAGCACAGAGTGGAAGGCGCA -3' | 3L: 13222721.. 13223494 |
| dco | Interband 100B3/ 100B4-5 | Part of the first intron (in the 5’ noncoding region) of the *discs overgrown (*[*CG2048*](http://flybase.org/cgi-bin/gbrowse/dmel/?dontadjust=1&name=FBgn0002413)*)* gene | 5'- GCGTCATTGCATTCGGCTGTGT -3' 5'- AGCGATCGAAATGCGTGAGCG -3' | 3R: 26884775.. 26885401 |
